# Cranberry polyphenols and agave agavins impact gut immune response and microbiota composition while improving gut barrier function, inflammation, and glucose metabolism in mice fed an obesogenic diet

**DOI:** 10.3389/fimmu.2022.871080

**Published:** 2022-08-16

**Authors:** Ana-Sofía Medina-Larqué, María-Carolina Rodríguez-Daza, Marcela Roquim, Stéphanie Dudonné, Geneviève Pilon, Émile Levy, André Marette, Denis Roy, Hélène Jacques, Yves Desjardins

**Affiliations:** ^1^ Institute of Nutrition and Functional Foods (INAF), Laval University, Québec, QC, Canada; ^2^ School of Nutrition, Faculty of Agriculture and Food Sciences, Laval University, Québec, QC, Canada; ^3^ Department of Food Science, Faculté des sciences de l’agriculture et de l’alimentation (FSAA), Laval University, Québec, QC, Canada; ^4^ Laboratory of Microbiology, Wageningen University & Research, Wageningen, Netherlands; ^5^ Department of Plant Science, FSAA, Laval University, Québec, QC, Canada; ^6^ Department of Medicine, Faculty of Medicine, Cardiology Axis of Quebec Heart and Lung Institute, Laval University, Québec, QC, Canada; ^7^ Research Centre, Sainte- Justine Hospital, Montreal, QC, Canada

**Keywords:** polyphenols, agavins, innate and adaptive immunomodulation, *Ahr*, *Tlr2*, *Nlrp6*, gut microbiota, *Akkermansia muciniphila*

## Abstract

The consumption of plant-based bioactive compounds modulates the gut microbiota and interacts with the innate and adaptive immune responses associated with metabolic disorders. The present study aimed to evaluate the effect of cranberry polyphenols (CP), rich in flavonoids, and agavins (AG), a highly branched agave-derived neo-fructans, on cardiometabolic response, gut microbiota composition, metabolic endotoxemia, and mucosal immunomodulation of C57BL6 male mice fed an obesogenic high-fat and high-sucrose (HFHS) diet for 9 weeks. Interestingly, CP+AG-fed mice had improved glucose homeostasis. Oral supplementation with CP selectively and robustly (five-fold) increases the relative abundance of *Akkermansia muciniphila*, a beneficial bacteria associated with metabolic health. AG, either alone or combined with CP (CP+AG), mainly stimulated the glycan-degrading bacteria *Muribaculum intestinale*, *Faecalibaculum rodentium*, *Bacteroides uniformis*, and *Bacteroides acidifaciens.* This increase of glycan-degrading bacteria was consistent with a significantly increased level of butyrate in obese mice receiving AG, as compared to untreated counterparts. CP+AG-supplemented HFHS-fed mice had significantly lower levels of plasma LBP than HFHS-fed controls, suggesting blunted metabolic endotoxemia and improved intestinal barrier function. Gut microbiota and derived metabolites interact with the immunological factors to improve intestinal epithelium barrier function. Oral administration of CP and AG to obese mice contributed to dampen the pro-inflammatory immune response through different signaling pathways. CP and AG, alone or combined, increased toll-like receptor (TLR)-2 (*Tlr2*) expression, while decreasing the expression of interleukin 1ß (ILß1) in obese mice. Moreover, AG selectively promoted the anti-inflammatory marker *Foxp3*, while CP increased the expression of NOD-like receptor family pyrin domain containing 6 (*Nlrp6*) inflammasome. The intestinal immune system was also shaped by dietary factor recognition. Indeed, the combination of CP+AG significantly increased the expression of aryl hydrocarbon receptors (*Ahr*). Altogether, both CP and AG can shape gut microbiota composition and regulate key mucosal markers involved in the repair of epithelial barrier integrity, thereby attenuating obesity-associated gut dysbiosis and metabolic inflammation and improving glucose homeostasis.

## Introduction

It is widely recognized that diet is an essential determinant of health, yet the contribution of its individual components is intricate and complex (1). For instance, the Western diet, characterized by high fat, high sugar, and low fiber content, is one of the major factors contributing to the etiology of societal chronic diseases (2). It is now clear that this unhealthy diet impacts metabolic responses and, at the outset, causes perturbations in the host–microbiota community structure, i.e., dysbiosis. Such an imbalance of the gut microbiota has harmful consequences on the intestinal barrier function and immune function. At first, the proliferation of opportunistic bacteria alters the integrity of the intestinal lining, creating the ideal conditions for their infiltration through the inner mucus layer and the gut epithelium, all of which increases mucosal inflammation and immune response (3). At this stage, bacterial endotoxins, such as lipopolysaccharide (LPS), eventually enter the portal circulation, leading to metabolic endotoxemia, systemic low-grade inflammation (4), and increased risks of cardiometabolic disease (2). The gut microbiota is thus a key intermediary underlying immunological processes leading to metabolic disorders and involves both innate and adaptive immune responses. Interestingly, certain dietary constituents can specifically shape the gut microbiota through a prebiotic action (5). Prebiotics are bioactive compounds from the diet that are selectively used by the gut microbiota as substrates conferring health benefits to the host (6). Currently, the category of prebiotics is dominated by non-digestible oligosaccharides (e.g., fructans, galactans, and resistant starch), but the concept of prebiotics has recently been expanded to include a variety of dietary ingredients like polyphenols. In fact, 90%–95% of dietary polyphenols reach the colon intact where they modulate the composition of the gut microbiota and are degraded to potentially bioactive microbial metabolites conferring health benefits (6).

Cranberry polyphenols from *Vaccinium macrocarpon* spp. exhibit prebiotic-like effects by stimulating the growth of beneficial bacteria and repressing the proliferation of pathobionts (7–9). As previously shown by our research group, the consumption of cranberry extracts rich in polyphenols, such as proanthocyanidin polymers (PACs), promotes the growth of commensal bacteria, like *Barnesiella* (9), *Akkermansia muciniphila* (8), *Lactobacillus*, and *Coriobacteriales*, while repressing others, like *Oscillibacter* (9), *Romboutsia*, *Ruminiclostridium*, and *Roseburia* (7). We have also shown that the modulation of the microbial community following cranberry consumption reduced metabolic disorders, improving obesity-induced dysbiosis (7, 8). Although these positive outcomes have been linked to the downregulation of inflammatory responses (8, 9), there is a paucity of reports on the immuno-modulatory effects of cranberry polyphenols.

Agavins from *Agave tequilana* spp. are branched oligomers of fructose recognized as prebiotics, owing to their strong microbiota modulatory effects (10–13). In a mouse model of diet-induced obesity, agavins restored gut microbiota homeostasis and improved cardiometabolic parameters. Recently, Huazano-García et al. (14) have shown that the addition of agavins to a high-fat (HF) diet induced important shifts in the gut microbiota composition, favoring a higher proportion of *Bacteroides*, *Prevotella*, and *Akkermansia* genera and decreasing that of *Oscillospira* and *Ruminococcus* (14). Agavin supplementation also attenuated HF-induced metabolic disturbances, reduced body weight gain (10, 15, 16), and regulated glycemia (10) and lipidemia (10, 15, 16). However, the effects of agavins on the modulation of the mucosal immune response have been less studied. *In vitro* studies, as well as an exploratory analysis in a clinical trial, have unveiled evidence of the involvement of agavins in the selective differentiation of T cells toward a T helper balance (17) and the increase in the levels of immunoglobulin A (IgA) in newborn infants (18). In view of the gap of knowledge on the immunomodulatory role of agave-derived neo-fructans, further studies are needed to assess the role of these molecules on the innate and adaptive immune responses of the colonic epithelium.

Interestingly, mixtures of phenolic compounds and fructans naturally occur in plant extracts and have synergistic action on metabolism (19, 20). As reviewed by Peshev and Van den Ende (19), polyphenols and fructans display prebiotic and immunomodulatory effects and are sensed by gut epithelium receptors. However, the synergistic effects of cranberry polyphenols and agavins in modulating both gut microbiota and immunological responses remain to be fully evaluated. The aim of the present work is thus to evaluate the impact of cranberry polyphenols and agavin supplementation, alone and in combination, on cardiometabolic risk factors, gut microbiota composition, metabolic endotoxemia, colonic histomorphology, and mucosal immune response in a mouse model of high-fat high-sucrose diet-induced obesity.

## Materials and methods

### Experimental design of animal study

The experimental procedures followed the guidelines of the Canadian Council on Animal Care and were approved by the animal care committee of the Sainte-Justine hospital, Montreal, QC, Canada. Sixty 6-week-old C57BL/6J male mice were randomly divided into five groups (12 mice per group) and single-housed in a controlled environment (one mouse per cage; 12/12-h light–dark) with free access to food and drinking water. The mice were kept in hanging cages and fed a standard chow diet for 1 acclimation week (week -1) (2018 Teklad global 18% protein rodent diet, Harlan Laboratories). The experimental design is illustrated in **Figure S1**. After acclimation and still under chow diet, all mice were pretreated for 1 week (week 0) with their corresponding supplements. After week 0, the two control groups were assigned one to a chow diet and the other to a high-fat and high-sucrose diet (HFHS) (65% fat, 15% protein, and 20% carbohydrate) and were fed daily by gavage with vehicle (water). The three other groups were assigned to HFHS diet and received daily either cranberry polyphenols (HF+CP), agavins (HF+AG), or the combination of cranberry polyphenols and agavins (HF+CP+AG) for 9 weeks (see **Figure S1**). Mice receiving the cranberry polyphenol extract (CP) were supplemented with a dose of 200 mg/kg of body weight (BW), and those receiving agavins (AG) were supplemented with 1 g/kg of BW; each dose corresponds to the equivalent of 100 g of fresh fruit and 5 g/day in human based on the US Food and Drug equivalent (21). Food intake was monitored three times a week. The BW gain was measured twice a week under non-fasting conditions. After 9 weeks of supplementation, an oral glucose tolerance test (OGTT) was performed. Fecal samples were collected for bacterial DNA extraction before the start of the treatments (week 0) and at the end of the intervention (week 9) and were immediately placed in dry ice and stored at -80°C. After 9 weeks of supplementation, mice were euthanized by cardiac puncture under anesthesia with isoflurane (2%–3%; 0.5–1.5 l/min). Adipose tissues, organs, and intestines were collected, weighted, and immediately immersed in liquid nitrogen or RNAlater (Invitrogen) for subsequent analysis. Frozen tissues were then stored at −80°C. Colon tissues were collected and immersed in Carnoy’s fixation solution buffered formalin 10% at room temperature, for histological examination.

### Characterization of cranberry extract

American cranberry (*Vaccinium macrocarpon* Aiton) hydroethanolic extracts were provided by Diana Food Canada (Quebec, Canada). The characterization of the polyphenol contents of the cranberry extract was performed according to the method described by Dudonné et al. (22) and is described in detail in Anhê et al. (8). The characterization of the proanthocyanidin (PAC) content in cranberry extract is shown in **Table S1**.

### Characterization of agavin extract

Agavin powder from *Agave tequilana Weber* var. Azul (Olifructine™) was provided by Nutriagaves de México S.A. de C.V. (Jalisco, Mexico). The Olifructine™ powder contained 23.8% of fructooligosaccharide (FOS) oligomers (DP <10) and 76.1% of FOS polymers (DP >10). The characterization of the polysaccharide size distribution was performed by molecular weight-exclusion chromatography on a Waters Ultrahydrogel DP column using standards of different molecular weights. The contents of glucose (1.06 ± 0.35 g/l), fructose (8.78 ± 0.42 g/l), and sucrose (5.28 ± 0.45 g/l) were determined by HPLC on an Aminex 42-C column using glucose, fructose, and sucrose reagent-grade standards (Sigma-Aldrich, St. Louis, MO, USA).

### Assessment of host metabolic parameters

An OGTT was performed after 9 weeks of treatment on overnight 12-h-fasted mice. Baseline blood samples were collected before the OGTT and immediately centrifuged (3,500 rpm, 10 min at 4°C). Then, dextrose solution (2 μl/g of 50% dextrose) was administered by gavage to the mice, and blood samples (60 μl) were drawn from the lateral saphenous vein and stored at -80°C until the assays. Glycemia was determined using an Accu-Chek glucometer (Bayer) 0, 15, 30, 60, 90, and 120 min after the glucose load. Mouse insulin was determined at 0, 15, 30, 60, 90, and 120 min after the glucose load using an ultrasensitive ELISA kit (ALPCO, Salem, USA). The insulin resistance index (HOMA-IR) was calculated using the formula HOMA-IR = [fasting glycemia (nmol/L)*fasting insulinemia (µU/mL)/22.5]. The positive incremental area under the curve (iAUC), up to 30 min (iAUC 0–30 min) and 120 min (iAUC 0–120 min) during OGTT, was calculated for glucose (mmol/l per min) and insulin (pmol/l per min) as described by Brouns et al. (23).

Liver triglyceride (TG) and cholesterol contents were extracted in a 2:1 (v/v) chloroform–methanol medium which allows the formation of a monophasic solvent system. Then, lipid concentrations were determined using commercial kits (Randox Laboratories, Crumlin, UK). Plasma lipopolysaccharide (LPS) concentrations were determined using a kit based on the limulus amoebocyte lysate (LAL) reaction (LAL Kit Endpoint QCL1000, Lonza, Switzerland). Plasma concentrations of lipopolysaccharide-binding protein (LBP) were measured using sandwich solid-phase enzyme-linked immunosorbent assay (ELISA) kits following the manufacturer’s instructions (Hycult Biotech Inc.). Plasma total and free cholesterol (TC), triglyceride (TG), and high-density lipoprotein cholesterol (HDL-c) concentrations were measured using enzymatic colorimetric assay kits (Boehringer Mannheim, Montreal, Canada). Fecal short-chain fatty acids (SCFA) were assessed by gas chromatography as previously described (24).

### Colon histomorphology analysis

Colon tissue preparation was performed as previously described (25). Briefly, pieces of PBS-flushed colon tissues were transferred into a tube containing cold methanol-Carnoy’s solution (60% methanol, 30% chloroform, and 10% glacial acetic acid). The samples were fixed for 3 h at 4°C. Once fixed, the tissues were washed with cold 70% ethanol and stored at 4°C until they were processed. Tissue processing was performed at the IBIS laboratory of Molecular Imaging and Microscopy, Laval University (Québec, QC, Canada). The samples were placed into a tissue cassette and subjected to a tissue processor for standard embedding in paraffin wax (Tissue-Tek VIP, Vacuum Infiltration Processor Sakura brand). Samples were treated with alcohol 95% for 45 min, alcohol 100% 3× for 45 min, toluene 2× for 45 min, and paraffin for 1 h 30 min, 2 h, and 4 h. Paraffin-embedded sections of colon tissues were stained with both periodic acid Schiff and Alcian Blue (AB-PAS). The number of mucous-secreting goblet cells (GC), mucus thickness, and crypt depths were quantified following the protocol previously reported by our research group (7).

### RNA isolation and transcription profiling using PCR array

Colon tissues stored in RNAlater (Invitrogen) at -80°C were thawed on ice, and total RNA was extracted using an RNeasy Mini Kit (Qiagen, #74104), according to the manufacturer’s instructions. During the RNA extraction, samples were DNase treated using the RNase-Free DNase kit (Qiagen; #79254). The RNA samples were assessed for concentration and quality using RNA NanoChips in Agilent 2100 Bioanalyzer (Agilent Technologies, Germany). RNA was deemed of sufficient quality when the RNA integrity number (RIN) was above 8. One microgram of RNA from individual animal samples was used for first-strand cDNA synthesis using the RT^2^ First Strand Kit (Qiagen/SABiosciences; #330401). One additional step for removing traces of genomic DNA was included in the cDNA reactions. Briefly, the reaction mixture to eliminate genomic DNA was incubated for 5 min at 42°C, then placed immediately on ice. To reverse transcribe, 10 µl of reverse-transcription mix was added to 10 µl of genomic DNA elimination mix. The mixture was incubated at 42°C for exactly 15 min then incubated at 95°C for 5 min. For each experimental condition, cDNA from individual animals were pooled by batches, corresponding to three cDNA samples per pool, resulting in four batches encompassing the 12 mice from each dietary group (n = 3/batch and four batches per group). The resultant cDNA products were analyzed by RT² Profiler Mouse Innate & Adaptive Immune Responses PCR Array (Qiagen #330231 PAMM-052ZA). The PCR array combines the quantitative performance of SYBR Green-based real-time PCR with the multiple-gene profiling capabilities of microarray. The plates of 96 wells contained gene-specific primer sets for 84 markers for adaptive and innate immune responses, including chemokines and cytokines, five housekeeping genes, and two negative controls. The thermocycling protocol was as follows: 95°C for 10 min for hot-start polymerase activation, followed by 40 cycles of denaturation at 95°C for 15 s, annealing at 60°C for 1 min, and a melting curve stage as default setting from 60°C to 95°C on an Applied Biosystems ABI 7500 Fast real-time cycling platform. Array quality was controlled by the following criteria: (1) the average Ct of the array built-in positive PCR control (PPC-well) was 20 ± 2, for PCR array reproducibility. Furthermore, no two arrays had an average PPC Ct >2 from each other, indicating that no amplification-inhibiting factors were present; (2) the average of the built-in reverse transcription control (RTC-well) was PPC ≤5, indicating no inhibition of reverse transcription, and (3) a Ct of genomic DNA control (GDC) >35, indicating no genomic DNA contamination. Stability of internal controls (housekeeping genes) was further evaluated by geNorm and NormFinder applications. The following reference genes were used for normalization: β-glucuronidase (*Gusb*), glyceraldehyde-3-phosphate dehydrogenase (*Gapdh*), and heat shock protein 90 alpha (cytosolic), class B member 1 (*Hsp90ab1*). Data were analyzed using the ΔΔCt method to determine the fold change of each gene normalized to the expression level of the reference genes.

### Gene expression analysis

Colon samples were processed as described above for the total RNA extraction, DNase treatment, and RNA integrity method and instrument. Then, 2 μg RNA was reverse-transcribed to cDNA using the High-Capacity cDNA Reverse Transcription Kit (Applied Biosystems, Carlsbad, CA) following protocol instructions. Resultant cDNAs were diluted 1:10 with nuclease-free water before amplification by quantitative real-time polymerase chain reaction (qRT-PCR). Amplification was performed on a ViiA 7 Real-Time PCR System (Thermo Fisher Scientific) using PowerUp SYBR^®^ Green Master Mix (Applied Biosystems, USA) on a 384-well reaction plate. Each sample was tested in duplicate. The primer pairs used for qRT-PCR amplification are presented in **Table S2**. Primers of internal reference genes were custom designed in compliance with MIQE guidelines and employing Geneious software (version 9.0). Reference genes were submitted to stability validation by geNorm software. The gene expression of the samples was then normalized against the three most stable reference genes, actin beta (*Actb*), peptidylprolyl isomerase B (*Ppib*), and hypoxanthine phosphoribosyltransferase (*Hprt*), and then calculated using the comparative Ct method (2−ΔΔCt).

### Fecal DNA extraction and 16S rRNA gene amplicon library preparation and sequencing

The genomic fecal DNA was extracted using a ZR Fecal DNA Kit (D6010; Zymo Research Corp., Orange, CA) following the instructions of the manufacturer. DNA concentration and quality were determined using an ND-1000 NanoDrop (NanoDrop Technologies, Wilmington, DE, USA). High-throughput sequencing was performed at the IBIS laboratory of Molecular Imaging and Microscopy, Laval University (Québec, QC, Canada). For library preparation, the 16S rRNA V3–V4 regions were amplified by using degenerate primers 341F (5′-CCTACGGGNGGCWGCAG-3′) and 805R (5′-GACTACHVGGGTATCTAATCC-3′). The primers were adapted to incorporate the transposon-based Illumina Nextera adapters (Illumina, USA) and obtain multiplexed paired-end sequencing. The 16S metagenomic fragments were purified using 35 µl of magnetic beads (AxyPrep Mag PCR Clean-Up Kit; Axygen Biosciences, USA) per 50-µl PCR reaction. Library quality control was analyzed with a Bioanalyzer 2100 using DNA 7500 chips (Agilent Technologies, USA). An equimolar pool was quality-checked and quantified using PicoGreen (Life Technologies, USA) and loaded on a MiSeq platform using 2 × 300-bp paired-end sequencing (Illumina, USA).

### 16S rRNA sequence processing

Demultiplexed RAW data files were treated and analyzed as previously published (7). Briefly, raw data were imported into R Studio software (version 3.6.1, R Core Team, Vienna, Austria). Primer sequences were removed from paired forward and reverse reads using Cutadapt (version 2.4) (26). Quality-filtered reads (scores >20) were then processed using the Divisive Amplicon Denoising Algorithm (DADA2) pipeline (27). A table for amplicon sequence variants (ASVs, a higher analog of operational taxonomic units—OTUs) was constructed, and taxonomy assignment was performed using the SILVA database (SILVA SSU Ref 132 NR, Dec 2017) as the reference dataset (28). The DECIPHER R package (29) was used to construct a phylogenetic tree from the ASV table. Singletons were removed. The *phyloseq* package in R (30) was used to obtain phylogeny-aware distances between microbial taxonomies. The α-diversity metrics, including Chao1, Shannon, and Simpson indices, were measured within dietary categories. Bray–Curtis dissimilarity and unweighted UniFrac of ASVs (OTUs) were calculated for β-diversity analysis and plotted through principal coordinate analysis (PCoA). Relative taxon abundances, at the phylum, family, and genus levels, were plotted using the *ggplot2* package (31).

### Microbial functional profiling

Predicted functional profiles were analyzed from the obtained ASV taxa table as previously described (7). ASV-related functions were inferred based on the Kyoto Encyclopedia of Genes and Genomes (KEGG) Ontology (KO) functional and pathway profiles. Functions were calculated using the Tax4Fun2 package (32). The ASV FASTA files with 97% similarity relative to the SILVA sequence database were aligned against the 16S rRNA reference sequences (“Ref100NR”) with copy number correction enabled by BLAST (version 2.9.0). The functional redundancy index (FRI), denoting the proportion of species fulfilling a specific function and their phylogenetic relationship between them (32), was also calculated using Tax4Fun2 software. We subsequently plotted these FRI values in a PCoA based on the Bray–Curtis distances and analysis of similarities (PERMANOVA). A high FRI is represented by the closeness of clusters of dietary groups and indicates that functions displayed by bacterial taxa making up the gut microbiota are similar among groups of mice, whereas a low FRI of the gut microbiota among groups is represented by a separated clustering of groups and suggests that a distinct functional profile has been detected. The functional predictions corresponded, on average, to 11% (102) of the 913-total assigned ASVs per sample, representing 34% of all sequences obtained.

### Microbial community composition and differential abundance statistical analysis

A Mann–Whitney U test or Kruskal–Wallis test with multiple-comparison correction according to the false discovery rate (FDR) method of Benjamini and Hochberg (Prism 8.0, GraphPad software, CA) was performed among dietary groups while measuring the α-diversity indices and differentially abundant features. Significance of sample dispersion (clusters) from PCoAs was calculated by stratified permutational multivariate analysis of variance (PERMANOVA) with 999 permutations using the *vegan* package in R (*p* < 0.05).

Differential abundance analyses of taxa between dietary groups were determined at the genus level using the *DESeq2* package in R (33). Fold changes of taxa were reported. Results found to be significant (*p* < 0.05 after multiple-hypothesis testing) were expressed as Log_2_-fold change in the HFHS-fed mice relative to chow-fed mice and the HFHS-fed groups treated with CP, AG, and CP+AG relative to untreated counterparts.

### Mouse phenotype statistical analysis

One-way ANOVA with a Dunnett *post-hoc* test (GraphPad Prism 8.0) was performed between dietary groups relative to HFHS control, for individual pairwise comparisons of data from mouse metabolic measurements and histological analysis. Particularly, two-way repeated-measurement ANOVA models with Dunnett’s *post-hoc* test correction were used for data analysis from weekly body weight gain and OGTT. Array data were analyzed using one-way ANOVA if the samples passed the normality analyses by using RT^2^ Profiler PCR Array Data. *p* values less than 0.05 were considered statistically significant whereas *p*-values between 0.05 and 0.1 were considered as showing a trend. Results were expressed as means ± standard error of the mean (SEM). Gene expression data were processed by an analysis of variance of normalized data by a one-way ANOVA with Dunnett *post-hoc* test (GraphPad Prism 8.0).

## Results

### Cranberry polyphenols with agavins reduced visceral body fat mass depot in HFHS-diet-induced obese mice

**Figure 1:**
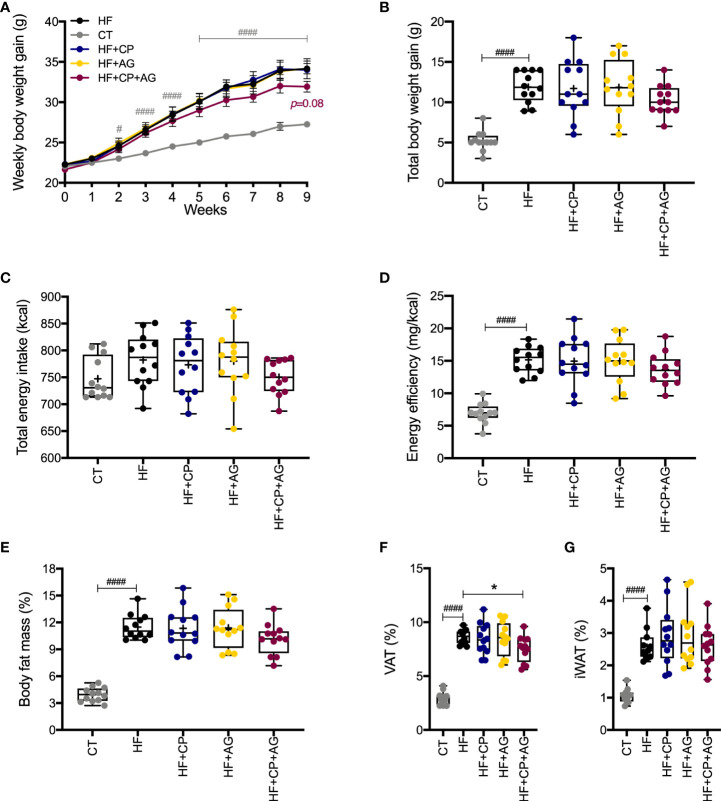
Cranberry polyphenol + agavin effects in body weight gain and body fat mass in HFHS-fed mice. **(A)** Weekly body weight gain expressed in grams gained by male C57BL/6 mice consuming the indicated diets for the 9-week intervention period. **(B)** Total body weight gain in grams at the end of week 9. **(C)** Total energy intake in kilocalories (kcal). **(D)** Energy efficiency corresponding to the ratio of body weight in milligrams (mg) to energy intake in kilocalories (mg/kcal). **(E)** Percentage (%) of body fat mass expressed relative to body weight at 9 weeks. **(F)** Percentage (%) of visceral adipose tissue (VAT%), represented by the sum of retroperitoneal white adipose tissue (rpWAT) + epididymal white adipose tissue (eWAT). **(G)** Percentage of inguinal white adipose tissue (IWAT%). CT — chow diet. HF — high-fat high-sugar diet. HF+CP — high-fat high-sugar diet and cranberry polyphenols. HF+AG — high-fat high-sugar diet and agavins. HF+CP+AG — high-fat high-sugar diet and the combination of CP+AG. Two-way repeated-measure ANOVA (RM two-way ANOVA) with Dunnett’s multiple-comparison test (*post-hoc* test) was employed to calculate the significance between groups at different time points. One-way ANOVA with a Dunnett’s multiple-comparison test (*post-hoc* test) was employed to calculate the significance of the differences between groups. Values are expressed as the mean ± SEM (n = 12). Boxplots represent the distribution of data with the mean represented by the mark “+” within the boxes, the median represented by the dark horizontal line and interquartile range by the box, *p < 0.05 as compared to the HFHS-control group. ^#^p < 0.05; ^###^p < 0.001. Chow-control group versus HFHS-control group.

Mice fed the HFHS diet gained more BW (*p* < 0.0001) and body fat mass (*p* < 0.0001) than their CT-fed counterparts after 9 weeks of dietary treatment with no difference in energy intake **(** **Figures 1A–G** **)**. However, the oral administration of CP or AG to mice fed HFHS did not affect BW gain. In addition, all mice on the HFHS-diet had a similar high proportion of fat mass, regardless of the CP or AG oral supplementation **(** **Figure 1E** **)**. In particular, no significant changes were observed in iWAT or VAT of obese HFHS-fed mice receiving either CP or AG, although the CP+AG-fed mice presented a decreased VAT proportion, as compared to non-supplemented HFHS (*p* < 0.05) (**Figure 1F**).

### Cranberry polyphenols and agavins ameliorate glucose homeostasis in HFHS-fed mice, without affecting lipid metabolism

**Figure 2:**
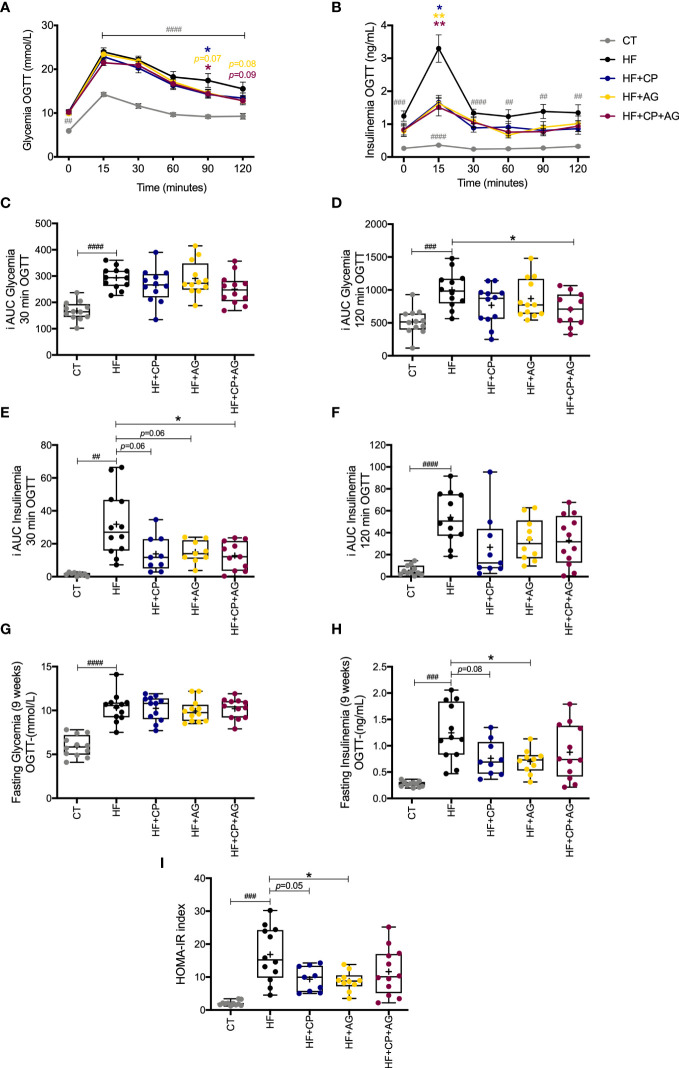
Effects of cranberry polyphenols (CP) and agavins (AG) in glucose in HFHS-diet induced obese mice. Mice were fed either a chow diet (CT) or high-fat high-sugar diet (HF); mice on an HF diet were supplemented with CP (HF+CP), AG (HF+AG), or the combination of both CP+AG (HF+CP+AG) for 9 weeks. Blood samples were collected at different time points during OGTT for glycemia and insulinemia measurements **(A–H)**. **(A)** Glycemia during OGTT (mmol/L). **(B)** Insulinemia during OGTT (ng/mL). **(C)** Mean plasma incremental area under the curve (iAUC) glucose 0–30 min OGTT. **(D)** Mean plasma (iAUC) glucose 0–120 min OGTT. **(E)** Mean plasma iAUC insulinemia 0–30 min OGTT. **(F)** Mean plasma iAUC insulinemia 0–120 min OGTT. **(G)** Fasting plasma glycemia levels during OGTT (mmol/L). **(H)** Fasting plasma insulinemia levels during OGTT (ng/mL). **(I)** Homeostasis model assessment of insulin resistance index (HOMA-IR). Two-way repeated-measure ANOVA (RM two-way ANOVA) with Dunnett’s multiple-comparison test (*post-hoc* test) was employed to calculate the significance between groups at different time points. One-way ANOVA with a Dunnett’s multiple-comparison test (*post-hoc* test) was employed to calculate the significance of the differences between groups. Values are expressed as the mean ± SEM. Boxplots represent the distribution of data with the mean represented by the mark “+” within the boxes, the median represented by the dark horizontal line and interquartile range by the box. *p < 0.05; **p<0.01 as compared to the HFHS-control group. ^##^p < 0.01; ^###^p < 0.001; ^####^p < 0.0001 chow-control group versus HFHS-control group.

The mice fed the HFHS diet for 9 weeks exhibited impaired glucose metabolism (**Figures 2A–I**). In particular, there were increases in glycemia and insulinemia at different time points (0, 15, 30, 60, 90, and 120 min) during the OGTT in HFHS-fed mice compared with CT-fed mice (**Figures 2A, B** **)**. After oral glucose load, glycemia iAUC up to 120 min and insulinemia iAUC up to 30 min were significantly reduced in the CP+AG fed mice as compared with the non-supplemented HFHS-fed group (*p* < 0.05). Mice fed the AG diet had lower levels of fasting plasma insulin relative to their non-supplemented HFHS counterparts (*p* < 0.05). HF+AG mice and those fed the HF+CP-diet also had lower HOMA-IR index values (HF+CP and HF+AG p < 0.05) compared to non-supplemented HFHS mice (**Figures 2H, I** **)**. Mice fed the HFHS diet had higher concentrations of liver TG, total and free cholesterol, and HDL-cholesterol (*p* < 0.0001 relative to CT) (**Figures S2A–E**). The CP and AG diets, given separately or in combination, did not significantly change any of the above-described lipid levels.

### Cranberry polyphenols and agavins distinctly influence the gut microbiota composition and predicted functions in HFHS-fed mice

After 9 weeks of dietary intervention, the bacterial richness and diversity, as shown by Chao1 and both Shannon and Simpson indices respectively, were significantly affected by the HFHS diet when compared to CT-fed control group (**Figure S3A**). However, no significant differences were observed in bacterial richness and diversity between groups following the CP, AG, and the combination of both diets relative to controls. Principal coordinate analysis (PCoA) was then performed to visualize the dissimilarities of gut microbiota composition between mice fed the different diets. To that effect, Bray–Curtis and weighted UniFrac were applied. In the PCoA plots, the gut microbiota composition of mice fed the HFHS diet clearly separated from those fed the CT control diet (**Figures S3B–E**). Among the treated groups, especially the mice fed the CP-containing diets clustered away from HFHS-fed mice, indicating that cranberry polyphenols mainly modified the taxonomic composition of the gut microbiota of obese mice. Permutational multivariate ANOVA (PERMANOVA), considering distance centroids of each dietary cluster, revealed significant differences in the overall gut microbiota composition between controls and CP-treated mice, administered either individually or in combination with AG at 9 weeks posttreatment (R^2^ = 0.54452, *p* = 0.001 *** based on the Bray–Curtis metric) (**Figure S3B**). Moreover, the PCoA plot based on weighted UniFrac, a metric based in a taxon-phylogenetic tree, confirmed the gut microbiota dissimilarities between non-supplemented HFHS versus CP- and AG-fed groups (**Figure S3C**); the PERMANOVA analysis revealed a R^2^ of 0.60617, *p* = 0.001***. The similarities found in the taxonomic structure of all HFHS-supplemented mice suggest a definite contribution of CP and AG, alone or in combination, in modifying the gut microbiota structure in a condition of obesity. Regarding functional divergences, Tax4fun2-predicted pathways of the gut microbiota as well as the microbial functional redundancy were visualized in a PCoA plot based on Bray–Curtis metric (**Figures S3D, E**). These predictive analyses indicated that CP and AG, when conjointly administrated to obese mice, may contribute the most to drive distinct functional pathways in the gut microbiota than when administrated individually. This finding was clearly reflected in a separated clustering of mice fed the CP+AG from HFHS control group in the PCoA plot based on functional redundancy (PERMANOVA R^2^ = 0.35397, *p =* 0.001***) (**Figure S3E**). On the contrary, the mice fed either the CP or AG overlapped on the PCoA, indicating that these dietary treatments, although fed individually, produced a similar functional structure in the gut microbiota of obese mice, yet they clustered distantly of HFHS-fed control.

### Cranberry-polyphenols selectively increase *A. muciniphila*, while agavins promote butyrate-producing bacteria in HFHS-fed mice

At the phylum level, the obesogenic diet significantly induced higher proportions of Firmicutes and Actinobacteria, along with a lower proportion of Bacteroidetes (recently, taxonomically named as Bacteroidota), as compared to CT-fed mice. On the contrary, the oral administration of CP, AG in HFHS-induced obese mice, or the combination of both prompted Bacteroidota and decreased Actinobacteria phyla as compared with their non-supplemented counterparts. Interestingly, only the obese mice fed the CP diet presented an increased proportion of Verrucomicrobia phyla compared to HFHS (*q* = 0.0768) (**Figure S4A**). At a deeper taxonomic level, *Muribaculaceae* and *Lactobacillaceae* families were drastically hampered by the HFHS-diet, while *Ruminococcaceae* and *Eggerthellaceae* were promoted as compared to the CT diet. Among these taxa, the relative proportion of *Eggerthellaceae* was significantly inhibited by CP and AG administered either individually or conjointly to HFHS-fed mice (*p <* 0.05). Surprisingly, only the mice receiving both CP+AG had a significantly reduced proportion of the opportunistic *Ruminococcaceae* family, favoring at the same time *Bacteroidaceae* (*p <* 0.05 vs. HFHS). Moreover, the *Akkermansiaceae* and *Bacteroidaceae* families were promoted in CP-fed mice, although these changes did not reach statistical significance after multiple comparison corrections (*q* = 0.0768 and *q* = 0.0847 vs. HFHS, respectively) (**Figure S4B**).

**Figure 3:**
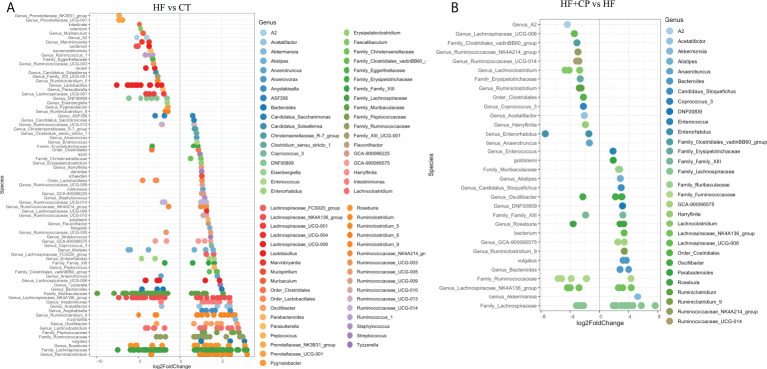
Taxa significantly upregulated and downregulated in mice fed high-fat high-sugar diet alone and in combination with cranberry polyphenols. **(A)** Differential abundance analysis (*DESeq2*) revealed 79 amplicon sequence variants (ASVs) significantly modulated in mice fed high-fat high-sugar diet (HF) relative to lean mice fed chow diet (CT). **(B)** 32 ASVs were significantly modulated in mice fed HF + cranberry polyphenols (HF+CP) relative to HF mice. ASVs (*p* < 0.05, FDR-corrected) are represented by single data points (with some data points overlapping) within each genus grouped on the x-axis, and by color fitting to which taxonomic family the ASVs belong. Data are plotted as log2 fold change. n = 12 mice per group. ASVs to the right of the zero line are more abundant, and ASVs to the left of the zero line are less abundant.

**Figure 4:**
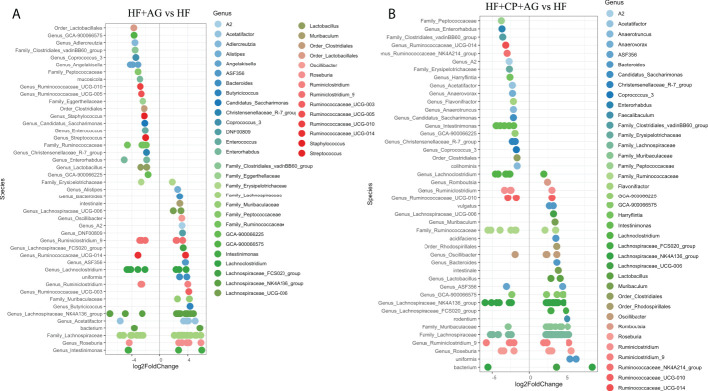
Taxa significantly upregulated and downregulated by agavins and the combination of agavins and cranberry polyphenols in high-fat high-sugar-fed mice. **(A)** Differential abundance analysis (*DESeq2*) revealed 45 amplicon sequence variants (ASVs) significantly modulated in mice fed HF + agavins (HF+AG) relative to HF. **(B)** Forty-four ASVs were significantly modulated in mice fed HF + cranberry-polyphenols with agavins (HF+CP+AG) relative to HF mice. ASVs (*p* < 0.05, FDR-corrected) are represented by single data points (with some data points overlapping) within each genus grouped on the x-axis, and by color fitting to which taxonomic family the ASVs belong. Data are plotted as log2 fold change. n = 12 mice per group. ASVs to the right of the zero line are more abundant and ASVs to the left of the zero line are less abundant.

Further *DESeq2-*differential analysis was performed to evaluate the taxonomic bacterial genera significantly inhibited or promoted by the CP, AG, or the combination of both diets relative to HFHS control diet. Only significant taxon abundances after multiple-testing correction (FDR-adjusted *p*-value, *q* < 0.05) having two-fold or higher changes are presented. The HFHS diet significantly hampered taxa that were characterizing the gut microbiota of lean mice fed the CT diet. This is the case of the carbohydrate-degrading *Genus_Prevotellaceae_NK3B31_group* and *Genus_Prevotellaceae_UCG-001*; their proportions were lowered by a robust eight-fold in HFHS-fed mice. Likewise, butyrate-producing species *Muribaculum intestinale*, *Faecalibaculum rodentium*, and the *Lactobacillus* genus were inhibited at least five-fold in obese mice as compared to those fed the CT diet. Conversely, some opportunistic taxa thrived under the HFHS diet, such as *Ruminiclostridium* and unassigned taxonomic genera belonging to *Lachnospiraceae*, *Ruminococcaceae*, and *Peptococcaceae* families (**Figure 3A**). Once the obese mice were fed the CP diet (**Figure 3B**), the proportions of the above opportunistic taxa, among others, were significantly reduced, while symbiotic species were favored, including *A. muciniphila*, *Genus_Bacteroides* (e.g., *Parabacteroides goldsteinii*), *Family_Muribaculaceae*, and *Genus_Alistipes*, being from two- to five-fold higher than in non-supplemented HFHS mice. On the other hand, AG supplementation, in addition to significantly suppressed species belonging to *Ruminococcaceae* and *Peptococcaceae* genus, favored the *M. intestinale*, *Roseburia*, *Butyricicoccus*, *Alistipes*, and *Muribaculaceae-*belonging species as compared to HFHS (**Figure 4A**). When CP+AG were conjointly administered to HFHS-fed mice, a larger number of opportunistic taxa that were increased in untreated obese mice were significantly lowered. In addition, the taxa *Lactobacillus*, *Bacteroides acidifaciens*, *F. rodentium*, and the genus *Muribaculum*, among others, were significantly stimulated (**Figure 4B**).

### Agavins consumption increases the fecal levels of butyric acid in HFHS-fed mice

The consumption of HFHS diet significantly decreased the fecal levels of the major SCFA acetic, propionic, and butyric acids at 9 weeks posttreatment, relative to control CT diet (**Figures S5A–C** **)**. Interestingly, the oral administration of AG counteracted the shift in butyric acid, significantly increasing its proportion (*p* < 0.05 vs. HFHS) (**Figure S5C**). However, this change was not significantly reproduced when AG was conjointly administrated with CP to HFHS-diet induced obese mice.

### Cranberry polyphenols and agavins improve the colonic mucus thickness in HFHS-diet induced obese mice

**Figure 5:**
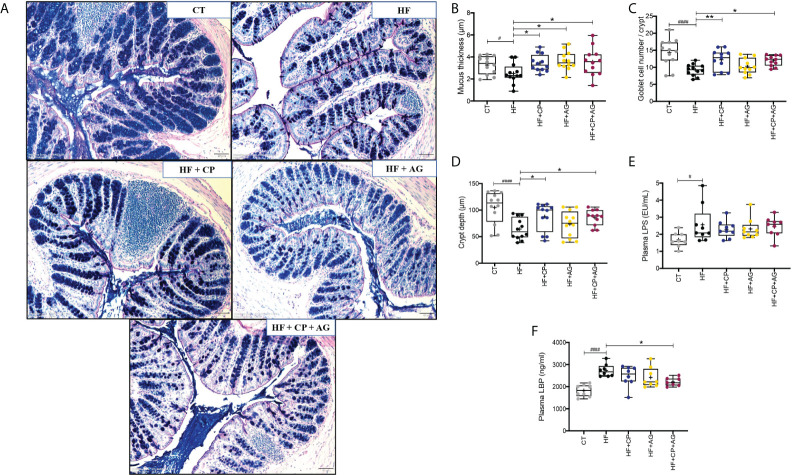
Cranberry polyphenols and agavins improved colonic mucus thickness and mucin-secreting goblet cell number, and also reduced circulating levels of lipopolysaccharide-binding protein in HFHS-diet induced obese mice. **(A)** The effect of HFHS-diet (HF) and the supplementation with cranberry polyphenols (CP), agavins (AG), and CP+AG were studied in representative histological images of the colon tissues of mice. A combination of Alcian Blue and periodic acid–Schiff staining (AB/PAS) was used to distinguish acidic (dark blue) and neutral (red) mucins. A purple color indicates the presence of both acidic and neutral mucins. Images shown are representatives of examined mice (n = 12) in each group. Images were taken using objective lens UPLSAPO 20×/0.75, magnification 12.6×, scale 50 μm. Histological parameters were evaluated in cross sections of colon tissues stained with AB/PAS staining: **(B)** mucus thickness (μm); **(C)** total goblet cell (GC) number per μm of crypt; **(D)** crypt depth (μm). **(E)** Plasma LPS (EU/mL); **(F)** plasma LBP (ng/ml). Ordinary one-way ANOVA with a Dunnett’s multiple-comparison test (*post-hoc* test) was employed to calculate the significance of the differences between groups. Boxplots represent the distribution of data with the mean represented by the mark “+” within the boxes, the median represented by the dark horizontal line and interquartile range by the box, *p < 0.05, **p < 0.01, as compared to HFHS-control group. ^#^p < 0.05 and ^####^p < 0.0001 chow-control group versus HFHS-control group.

Images of representative samples of colon tissues treated with AB-PAS staining showed that the CT fed mice presented an intense purple mucus layer closely associated with the epithelium and secreted mucus dispersed throughout the luminal space (**Figure 5A**). This mucus layer was drastically reduced when mice were fed the HFHS diet compared to CT. Conversely, CP and AG, administered individually or in combination, significantly restored the mucus thickness, as compared to their non-supplemented counterparts (**Figure 5B**). These findings are consistent with an increased mucin-secreting goblet cell number per colonic crypt, yet this was exclusively induced by polyphenols containing diets as observed in mice fed the CP or both CP+AG (*p* < 0.01 and *p* < 0.05 vs. HFHS, respectively) (**Figure 5C**). Moreover, the crypt depth was significantly increased in the mice receiving CP, AG, or both than in the untreated obese mice (**Figure 5D**).

### Cranberry polyphenols with agavins significantly improve metabolic endotoxemia in HFHS-fed mice

Lipopolysaccharide (LPS), an endotoxin released by opportunistic Gram-negative bacteria, can induce a local pro-inflammatory response in the gut depending on the type of LPS then permeate the mucosa and reach circulation where it binds to lipopolysaccharide-binding protein (LBP). Blood LBP levels were measured as a surrogate marker of metabolic endotoxemia, inflammation, and alteration of the intestinal barrier. The HFHS diet significantly increased plasma LPS levels (*p* < 0.05 vs. CT), together with the plasma LBP levels (*p* < 0.0001 vs. CT) (**Figures 5E, F** **)**, as indicative of chronic low-grade inflammation (endotoxemia) associated with a dysbiotic gut microbiota composition. While the consumption of the supplements did not affect plasma LPS levels, it did influence plasma LBP levels. Interestingly, mice fed both CP+AG, but not those fed CP or AG separately, exhibited reduced plasma LBP as compared to non-supplemented HFHS mice (*p* < 0.05) (**Figure 5F**).

### Cranberry polyphenols and agavins attenuate, in a different manner, colonic inflammatory responses of mice fed an obesogenic diet

**Figure 6:**
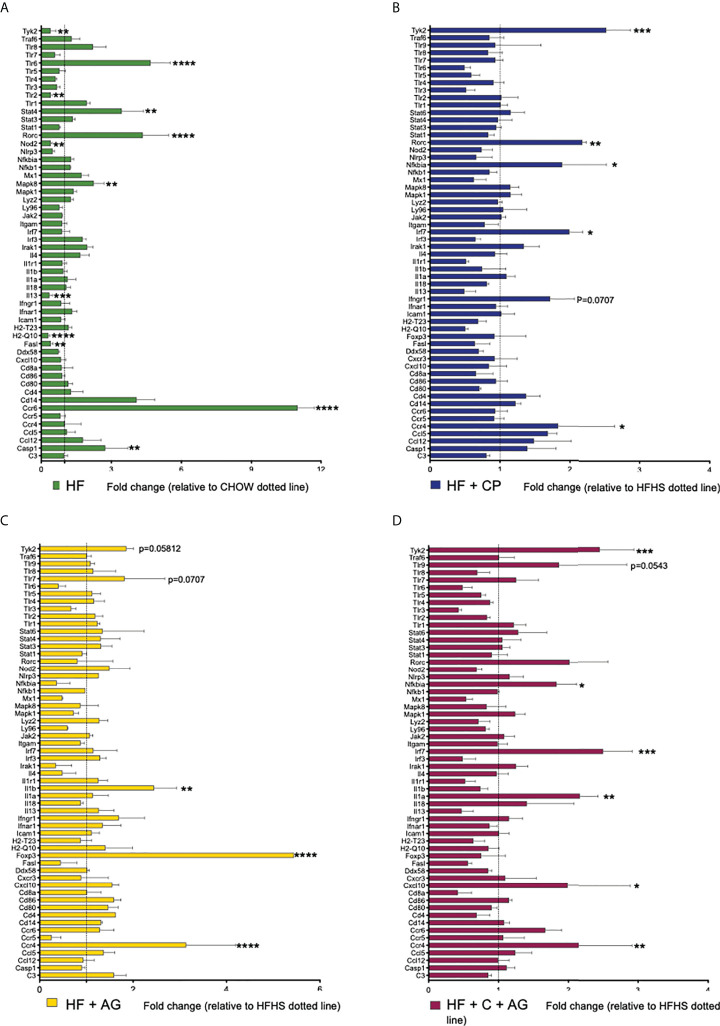
Effects of cranberry polyphenols (CP) and agavins (AG) in the modulation of innate and adaptive immune markers in HFHS-diet induced obese mice. An analysis of the expression profile of 84 genes associated with adaptive and innate immunity was performed in colon samples by microarray technology (Qiagen #330231 PAMM-052ZA). Mice were fed either a chow diet (CT) or high-fat high-sugar diet (HF); mice on an HF diet were supplemented with CP (HF+CP), AG (HF+AG), or the combination of both CP+AG (HF+CP+AG) for 9 weeks. Fold change of the panel of genes expressed in **(A)** HF relative to CT; **(B)** HF+CP relative to HF; **(C)** HF+AG relative to HF; and **(D)** HF+CP+AG relative to HF. The control HFHS group is displayed as a dotted line normalized to 1.0. Fold change to the right of the dotted line represents upregulation, and fold change to the left of the dotted line represents downregulation. Fold change was calculated using the ΔΔCt method; each gene was normalized to the expression level of the reference genes: glyceraldehyde-3-phosphate dehydrogenase (*Gapdh*), glucuronidase beta (*Gusb*), and heat shock protein 90 alpha (cytosolic), class B member 1 (Hsp90ab1). Two-way ANOVA was performed to calculate the significance of the differences between groups. *p <  0.05, **p <  0.01, ***p <  0.005, ****p < 0.0001.

The analysis of the expression profiles of genes associated with adaptive and innate immunity of colon samples revealed significant differences between HFHS and CT mice, along with differences between HFHS and all treated groups (**Figure 6** **;** **Table 1** **)**. As expected, the HFHS diet provoked a pro-inflammatory response in the colonic mucosa. Notably, genes related to a pro-inflammatory response, such as *Tlr-6* (*p* < 0.0001), *Mapk8* (*p* < 0.01), *Stat4* (*p* < 0.01), *Ccr6* (*p* < 0.0001), and *Casp1* (*p* < 0.01), were upregulated (**Figure 6A**). This pro-inflammatory response was attenuated in mice receiving CP, AG, or the combination of both diets (**Figures 6B–D**). On the one hand, CP stimulated the expression of *Tyk2* (*p* < 0.001), *Rorc* (*p* < 0.01), *Nfkbia* (*p* < 0.05), *Irf7* (*p* < 0.05), and *Ccr4* (*p* < 0.05) (**Figure 6B**). On the other hand, AG-diet upregulated anti-inflammatory markers such as *Foxp3* (*p* < 0.0001) (**Figure 6C**). Furthermore, when both CP and AG were fed conjointly, *Nfkbia* (*p* < 0.05), *Irf7* (*p* < 0.001), *Il-1α* (*p* < 0.01), *Tyk2* (*p* < 0.001), and *Cxcl10* (*p* < 0.05) were upregulated in HFHS diet-induced obese mice (**Figure 6D**).

**Table 1:** PCR array results for the high-fat control group vs. treated groups.

Refseq	Gene	Fold change	*p*-value	Gene description
				**HF vs. chow**
NM_011604	*Tlr-6*	3.68	<0.0001	Toll-like receptor (TLR)-6
NM_011487	*Stat4*	2.45	0.002	Signal transducer and activator of transcription 4
NM_011281	*Rorc*	3.35	<0.0001	RAR-related orphan receptor gamma
NM_016700	*Mapk8*	1.24	0.021	Mitogen-activated protein kinase 8
NM_009835	*Ccr6*	9.99	<0.0001	Chemokine (C–C motif) receptor 6
NM_009807	*Casp1*	1.74	0.0013	Caspase 1
NM_009841	*Cd14*	3.08	<0.0001	CD14 antigen
				**HF + CP vs. HF**
NM_018793	*Tyk2*	1.52	<0.001	Tyrosine kinase 2
NM_011281	*Rorc*	1.18	0.005	RAR-related orphan receptor gamma
NM_010907	*Nfkbia*	0.89	0.025	Nuclear factor of kappa light polypeptide gene enhancer in B-cells inhibitor, alpha
NM_016850	*Irf7*	0.99	0.012	Interferon regulatory factor 7
NM_009916	*Ccr4*	0.83	0.036	Chemokine (C–C motif) receptor 4
				**HF + AG vs. HF**
NM_008361	*Il-1β*	1.44	0.0014	Interleukin 1 beta
NM_054039	*Foxp3*	4.44	<0.0001	Forkhead box P3
NM_009916	*Ccr4*	2.14	<0.0001	Chemokine (C–C motif) receptor 4
				**HF + CP + AG vs. HF**
NM_018793	*Tyk2*	1.45	0.0005	Tyrosine kinase 2
NM_010907	*Nfkbia*	0.83	0.05	Nuclear factor of kappa light polypeptide gene enhancer in B-cells inhibitor, alpha
NM_016850	*Irf7*	1.50	0.0003	Interferon regulatory factor 7
NM_010554	*Il-1α*	1.17	0.0093	Interleukin 1 alpha
NM_021274	*Cxcl10*	0.99	0.0175	Chemokine (C–X–C motif) ligand 10
NM_009916	*Ccr4*	1.15	0.0059	Chemokine (C–C motif) receptor 4

### Cranberry polyphenols and agavins upregulate *Ahr*, *Nlrp6*, *Tlr-2*, and *Muc2* markers associated with improved epithelial barrier and reduced gut inflammation

**Figure 7:**
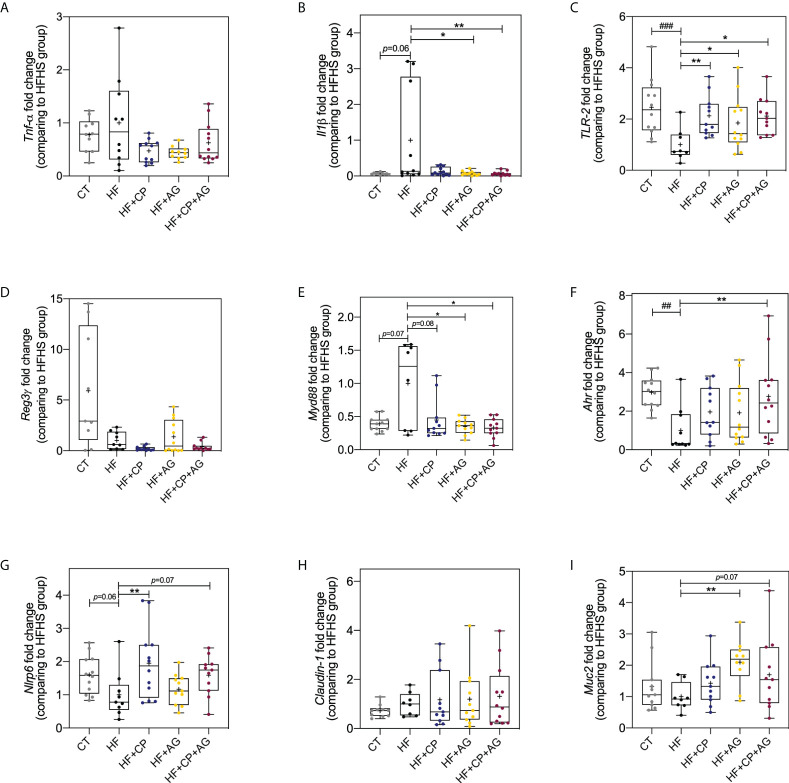
Effects of cranberry polyphenols and agavins in the relative expression of genes linked to the control of gut epithelial barrier function and inflammation. The fold change of colonic gene expression relative to HFHS-fed mice (normalized to 1.0) of **(A)** tumor necrosis factor-alpha (*Tnfα)*; **(B)** interleukin 1 beta (*Il1β)*; **(C)** Toll-like receptor 2 (*Tlr2*); **(D)** regenerating islet-derived protein 3 gamma (*Reg-3-γ*); **(E)** myeloid differentiation primary response 88 (*Myd88*); **(F)** aryl hydrocarbon receptor (*Ahr*); **(G)** NOD-like receptor family pyrin domain containing 6 (*Nlrp6*); **(H)** Claudin-1; **(I)** Mucin 2 (*Muc2*). Relative gene expression was determined by RT-qPCR. Fold change was calculated using the ΔΔCT method; each gene was normalized to the expression level of the reference genes actin beta (*Actb*), peptidylprolyl isomerase B (*Ppib*), and hypoxanthine phosphoribosyltransferase (*Hprt*). Mice were fed either a chow diet (CT) or high-fat high-sugar diet (HF); mice on a HF-diet were supplemented with CP (HF+CP), AG (HF+AG), or the combination of both CP+AG (HF+CP+AG) for 9 weeks. Boxplots represent the distribution of data with the mean represented by the mark “+” within the boxes, the median represented by the dark horizontal line and interquartile range by the box. Ordinary one-way ANOVA was performed to calculate the significance of the differences between groups. *p <  0.05, **p <  0.01 compared to the HFHS-control group. ^##^p < 0.01; ^###^p < 0.001 chow-control group versus HFHS-control group.

The beneficial effects of the dietary administration of CP, AG, or both on mucosal immunomodulation and barrier function were further confirmed by RT-qPCR gene expression analysis of *Tnfα*, *Il-1ß*, *Nlrp6*, *Reg3γ*, *Ahr*, *Tlr2*, *Claudin-1*, *Muc2*, and *MyD88* in colon tissues (**Figure 7**). As expected, the HFHS diet significantly downregulated *Nlrp6* inflammasome (*p* < 0.05), *Tlr2* (*p* < 0.001), and *Ahr* (*p* < 0.01) (**Figures 7C, F, G**). Noteworthily, CP selectively increased the expression of *Nlrp6* (*p* < 0.01) and *Tlr2* (*p* < 0.01) **(** **Figures 7C, G** **)**. AG also increased the expression of *Tlr2* (*p* < 0.05), and it exclusively upregulated *Muc2* (*p* < 0.01) and downregulated *MyD88* (*p* < 0.05) (**Figures 7C, E, I**). Although *Il-1ß* was initially found to be increased by AG from the microarray gene expression, further RT-qPCR analysis showed a significant downregulation of this gene. As a matter of fact, in addition to modulating the above anti-inflammatory markers, only the consumption of both CP+AG significantly lowered the expression of the inflammatory *Il-1ß* (*p* < 0.05) and stimulated *Ahr* in HFHS-fed obese mice (*p* < 0.01) (**Figures 7B, F** **)**. No significant differences were observed on *Tnfα*, *Reg3γ*, and *Claudin-1* colonic expression among groups.

## Discussion

### Role of polyphenols and agavins in improving cardiometabolic risk in obese mice

Consumption of a Western-type diet, rich in fat and sugars, induces gut microbiota dysbiosis, an imbalance between microbial commensals and opportunistic bacteria leading to gut barrier function disturbances, LPS translocation, and ultimately, intestinal immunological dysfunction. Together, these conditions promote the establishment of a chronic low-grade inflammation state (34). The present study aimed to investigate the impact of two different prebiotic ingredients, cranberry polyphenols and agavins, alone or in combination, on host cardiometabolic health, gut microbiota composition, gut epithelium histomorphology, and mucosal immunomodulation. We demonstrated that the oral supplementation with dietary realistic levels of cranberry polyphenols and agavins modified the gut microbiota composition, reinforced the colonic epithelial barrier, and attenuated gut inflammation in diet-induced obese mice. Both prebiotics have been previously shown to attenuate cardiometabolic disturbances typically observed in mice fed an obesogenic diet (10, 11). In the present study, CP and AG did improve glucose tolerance when conjointly administrated to HFHS-fed obese mice despite the lack of significant effects on weight loss and serum lipids. This last finding is in agreement with that reported by Tan et al. (35), who demonstrated an improved glucose homeostasis in obese mice co-supplemented with the polyphenol isoquercetin and the polysaccharide inulin. In addition, the dietary administration of CP, AG, and their combination significantly triggered the first phase of insulin secretion after oral glucose load, suggesting that these prebiotics synergically contribute to protect ß-cell function in diet-induced obesity (36).

A key factor contributing to the development of cardiometabolic diseases is diet-induced metabolic endotoxemia (4). Interestingly, the intake of a cranberry extract in combination with isomalto-oligosaccharides has been found to improve metabolic endotoxemia (37), as shown herein by lowering LBP plasma levels in HFHS-fed obese mice supplemented with the combination of CP and AG. Conversely, the individual oral supplementations either with CP or with AG did not lead to an LBP reduction, which is in opposition to previous interventions using either polyphenols or agavins alone (8, 11). Plasma LPS levels were less conclusive than those of LBP. It is important to bear in mind that it is well known that LPS is evanescent and that it can possibly interact with antibodies, antimicrobial peptides, macrophages, and even proanthocyanidins (PACs), making its measurement difficult (38, 39). Moreover, the type of LPS and its degree of lipid A acylation, dictated by the nature of the contributing Gram-negative bacteria, can also drive different metabolic outcomes (40). In this context, the LBP appears to be a more reliable marker of endotoxemia.

### Role of polyphenols and agavins in attenuating gut microbiota dysbiosis of HFHS-diet-induced obese mice

The prebiotic compounds tested impacted the gut microbiota composition but did not influence gut microbial diversity. The inferred gut microbial function also showed a dissimilar distribution in the PCoA, although these functional profiles remain to be further studied by transcriptomic and metagenomic approaches. The lack of effects on alpha diversity is somehow surprising and contrasts with the results of similar works. For example, Huazano-García et al. (11) observed a significant reduction in richness and diversity related to agavin consumption, while Rodríguez-Daza et al. (7) reported an increased microbial richness due to the consumption of cranberry polyphenols in a murine model of diet-induced obesity. However, changes in the microbial diversity are not a consistent feature of obesity (41). Indeed, diet-induced changes in the relative proportion of bacterial taxa mainly affect the ß-diversity and function of the gut microbiota. As expected, the intake of an obesogenic or a chow diet resulted in a large difference in bacterial communities, and the oral supplementation with the prebiotics CP and AG successfully modified the structure and the predicted function of the obese mouse gut microbiota. In line with this, a distinct clustering of CP+AG-fed mice away from those fed the HFHS was observed in the PCoA plots of the microbiota structure and inferred functional redundance.

The obesogenic diet provoked an imbalance in the proportions of the two major gut bacterial phyla Firmicutes and Bacteroidota, as reported elsewhere (42). However, both CP and AG, fed individually or combined, contributed to a rise of Bacteroidota, a predominantly glycan forager phylum usually associated with a lean microbiota (43). Analysis of the gut microbiota composition at the genus level uncovered bacterial species that were differentially promoted by polyphenols and agavins in obese mice. This is the case for the anti-obesity gut symbiont *A. muciniphila* (Verrucomicrobia phylum), which was selectively and robustly stimulated by five-fold in cranberry polyphenol-fed obese mice. It is indeed well established that *A. muciniphila* is negatively impacted by HFHS (44) and that dietary factors, especially polyphenols, which favor the growth of the bacteria, lead to reduced inflammation, improved insulin sensitivity, and decreased hyperinsulinemia during OGTT in HFHS fed mice (8). We also observe a tight relation between the presence of this bacteria in mice consuming CP and an improved glycemic response. Intriguingly, the polyphenol-induced bloom of *Akkermansia* in obese mice was abolished when agavin was supplemented. The selective prebiotic effects of polyphenols on *Akkermansia* were expected, as previously observed by our group (7, 8, 45). We further reported that the increase of this mucin-degrader bacterium is favored by the supplementation of oligomeric PACs (45), a class of polyphenols composed of polymers and oligomers of flavan-3-ols, largely present in our cranberry extract (22). There is no conclusive explanation for this observation, however, considering the antimicrobial action of polyphenols on HFHS-induced opportunistic bacteria; we hypothesize that the *Akkermansia* increase results from a reduced microbial competitiveness in the colon, in addition to its capacity to resist a wide array of antibiotics (46). It is possible that the prebiotic effects of the agavin-derived neo-fructans could prevail over those exerted by cranberry polyphenols, affecting in turn the extent of the inhibitory actions of phenolic molecules in the gut microbiota of obese mice. Our results are comparable to those of Neyrinck et al. (47) who reported a reduced abundance of *A. muciniphila* following combined supplementation with chitin-glucan (an insoluble dietary fiber from the cell wall of fungi) and pomegranate polyphenols. *A. muciniphila* thrives under low-fiber diets and is somewhat reduced in the presence of a fiber-rich diet. Indeed, Desai et al. (48) demonstrated that *Akkermansia* carbohydrate-active enzymes (CAZyme) are impervious to changes of dietary fibers as only two genes are differentially expressed in the presence of fiber diet (glycoside hydrolase family 13 and glycoside hydrolase family 43). This limited response indicates that *A. muciniphila* do not readily shift substrate utilization. The study of the *in vivo* effect of other dietary fibers and prebiotics on *A. muciniphila* abundance still requires more attention.

Several potential opportunistic gut bacteria observed in obese mice were inhibited by both dietary treatment CP and AG individually or combined, albeit to a different extent. One example is the family *Ruminococcaceae*, from which the genus *Ruminiclostridium* was inhibited four-fold in proportion by cranberry polyphenols. This is consistent with Rodriguez-Daza et al. (7) who observed a reduced relative proportion of *Ruminiclostridium* in the gut microbiota of mice fed a HFHS diet receiving cranberry or blueberry polyphenols. This could be considered a beneficial effect, since a positive correlation of *Ruminiclostridium* with obesity-related features such as body weight gain, energy efficiency, and a decreased in the size of cecum was reported. Likewise, several *Ruminococcaceae*-belonging taxa (i.e., *Anaerotruncus*) have been associated with a reduced production of SCFA and were positively associated with the presence of LPS in feces and blood (49). In contrast, agavins reduced the abundance of some taxa enriched in non-supplemented HFHS-fed mice, such as *Enterococcus*, *Streptococcus*, *Ruminococcaceae_UCG010*, *Ruminococcaceae_UCG005*, and *Angelakisella.*

Overall, the CP+AG diet-induced changes in the gut microbiota can be divided into three groups, the effects mainly related to CP, the effects mainly related to AG, and the effects exclusively observed with the combination of CP with AG. It is suggested that the gut microbiota modulation was most likely related to a specific dietary treatment when such an effect was observed simultaneously after the combination of both prebiotics and after the individual consumption of one, but not the other. For example, *Lactobacillus* spp., *Parabacteroides goldsteinii*, and *Bacteroides vulgatus* appear to be mainly stimulated by cranberry polyphenols, since this effect disappeared when agavins were solely consumed. Given the structural complexity of the agavin molecules (fructo-oligosaccharides combining linkages ß 2-1 and ß 2-6 with branching) (13), their consumption chiefly contributed to stimulate other *Bacteroides-*glycan-degrading bacteria (43). Surprisingly, AG-fed mice did not favor the growth of *Lactobacillus*, despite the fact that agavins have been reported to induce bifidogenic and lactobacilli effects (10). However, at the species level, we observed that agavins either alone or combined with polyphenols prompted *Bacteroides uniformis*, an important glycolytic bacterium with the largest repertoire of carbohydrate-active enzyme (CAZy) genes among all the *Bacteroides* species (50). Likewise, we noted a significant increase of *Muribaculum intestinale*, a bacterium belonging to the *Muribaculaceae* family (formerly known as *S24-7*), another group of bacteria recognized as complex carbohydrate degrader, stimulated in AG and CP+AG-fed mice. These results agree with the observations of Huazano-García et al. (11) using agavins, as well as with those of Li et al. (51) using highly polymerized inulin from chicory. Interestingly, Huazano-García et al. (14) reported that agavin supplementation stimulated both *B. uniformis* and *M. intestinale* while improving insulin resistance. We observed similar improvement of HOMA-IR in AG-fed mice. Since agavins are soluble fibers, it is not surprising that AG-fed mice, but not CP-fed mice, experienced an increase in the abundance of these bacteria.

As expected, the agavin-containing diet enriched SCFA-producing bacteria, like the genera *Butyricicoccus*, *Faecalibacterium*, and *Muribaculum.* Accordingly, butyrate production increased in the group of mice receiving AG. Interestingly, a higher proportion of *Muribaculum* spp. has been positively correlated with SCFA in C57BL/6 J obese mice fed inulin with different degrees of polymerization. Particularly in that study, this genus was prompted two-fold more by long-chain inulin than by short-chain inulin in HFHS-fed obese mice (51). This result indicates that *M. intestinale*, a species mainly accounting for the increased *M.* spp proportion, is specialized in degrading highly polymerized inulin, as is the case of agavin-derived neo-fructans used in our work. In fact, the microbial degradation of agavins appears to provide ideal cross-feeding conditions to produce butyric acid. To this effect, Koenen et al. (13) and Huazano-García et al. (11) also reported an increase in the production of butyric acid by agavins in a dynamic *in vitro* model (TIM-2) and in a mouse model, respectively.

### Impact of polyphenols and agavins on mucosal innate and adaptive immunomodulation

The intestinal eubiotic condition is the result of balanced innate and adaptive immune interaction with the gut microbiota and dietary factors along the intestinal epithelium. Particularly, the mucosal immune system senses the gut microbiota through microbe-associated patterns (MAMPs), such as toll-like receptors (TLRs). For instance, an imbalance between pro- and anti-inflammatory immune responses can be triggered by colonization of gut opportunistic bacteria. In the present study, the HFHS diet-induced obese mice presented a chronic low-inflammation marked by increased levels of *Tlr6*, *Mapk8*, *Stat4*, *Ccr6*, and *Casp1*, contributing to the development of gut dysbiosis as compared to chow-fed counterparts. We demonstrated that the consumption of the prebiotics CP and AG contributes to maintaining the intestinal barrier function and to attenuating gut inflammation. Notably, HFHS-fed mice receiving CP, AG, or both (CP+AG) presented a significant activation of *Tlr2* signaling as compared to untreated obese mice. In addition, AG promoted the *Muc2* expression linked to a reinforcement of the mucus layer protecting the intestinal epithelium. An increased expression of *Tlr2* has been shown to improve mucus homeostasis and to prompt mechanisms of immune response to protect the host against bacterial infection (52, 53). In addition, the preservation of the intestinal barrier function prevented the translocation of bacteria (4), as evidenced by the low levels of LBP in the plasma of CP+AG-fed mice. Further analysis would be desirable to understanding the modulatory effects of our treatments on immune cells.

The immunomodulatory effects reported herein could be elicited by the bloom of specific bacteria, by the release of their metabolic products, or even by the bacterial surface polysaccharides (PSA), LPS, or outer membrane proteins. The complexity of all these possible interactions hinders the understanding of the molecular mechanisms responsible for the cross talk between the gut microbiota and mucosal immunity. In this context, *A. muciniphila*, a bacterium specifically induced after CP diet, is one of the best studied. It is acknowledged that a pili-like protein of *A. muciniphila* MucT (Amuc_1100) activates *Trl2* and induces the secretion of cytokines as IL-10 (54). This is consistent with the *Tlr2* upregulation in the colon of CP-fed mice. Likewise, the increased abundance of *Bacteroides uniformis* observed in mice fed AG and CP+AG may help to improve the production of macrophage cytokines in response to a pathogenic bacterial stimulus and to restore the capacity of dendritic cells to induce a T-cell response (55). We surmise that the increased relative abundance of *B. acidifaciens*, which thrived exclusively with the CP+AG diet, induces an anti-inflammatory activity. In support of this interpretation, this taxon has been previously associated with enhanced gut immune defense mechanisms, among others, promoting the development of gut-associated lymphoid tissue (GALT) and the production of immunoglobulin A (IgA) (56). Taken together, consumption of the prebiotics CP and AG, alone or in combination, displays anti-inflammatory and immunomodulatory properties.

As an additional marker of gut barrier function, we analyzed by RT-qPCR the colonic expression of *Nlrp6.* This is an innate immune receptor participating in the inflammasome formation, which contributes to the regulation of mucus secretion and the production of antimicrobial peptides, thus protecting the gut epithelium from the microbial colonization (57). We observed a significant increased expression of *Nlrp6* in CP-fed mice and a non-significant trend (*p* = 0.06) in CP+AG fed mice. These results corroborate a recent report by Radulovic et al. (58) who observed that *Nlrp6* was activated by a daily dose of 25 µg of the polyphenol apigenin, associated with protection against intestinal inflammation. Also, Wang et al. (59) demonstrated that a mulberry supplement containing 50 mg/kg of anthocyanin promoted the colonic *Nlrp6* expression, helping to preserve the goblet cell count in mice suffering sodium dextran sulphate (SDS)-induced colitis. *Nlrp6* is indeed induced by TLR ligands expressed on goblet cells, promoting mucus renewal as a mechanism of protection (57). Incidentally, the stimulation of *Nlrp6* expression by CP-containing diets is associated with an increase in the number of goblet cells per crypt and a thicker mucus layer, compared to the non-supplemented HFHS-fed mice.

In parallel, we noted a significant upregulation of the tyrosine kinase 2 (*Tyk2*) in mice fed CP-containing diets and in chow-fed lean mice. *Tky2* action is mainly on innate immunity and inflammation as a signal transducer and activator of the JAK/STAT protein family, triggering a chain of responses involved in the expression of antimicrobial peptides, attenuation of hyperinflammation, and intestinal epithelial cell regeneration (60).

While innate immune responses were only influenced by CP-containing diets, adaptive immune responses were influenced by the three dietary treatments, but through different ancillary pathways. **Table 1** summarizes the significant changes observed in obese mice after the consumption of each dietary treatment. Noteworthily, CP induced a concomitant upregulation of the chemokine (C–C motif) receptor 4 (*Ccr4*) and RAR-related orphan receptor gamma (*Rorc*), both involved in T-cell regulation (Th17 and Treg). Likewise, CP+AG diet appeared to induce a similar adaptive response, since this diet also upregulated *Ccr4* and *Rorc*, but the latter did not reach a significant level. AG alone also significantly induced *Ccr4*, in this case accompanied by an increased expression of the forkhead box P3 (*Foxp3*), related with the transcription of Treg. Indeed, Treg cells residing in the lamina propria of the colon are essential to maintaining tolerance toward commensal microbiota and are involved in the attenuation of inflammation (61). Interestingly, the upregulation of *Foxp3* has previously been associated with an increase in immunomodulatory interleukin-10 (IL-10) by inducing Treg lymphocytes (62). Given that the consumption of agavins has also been previously associated with an increase of IL-10 (11), the upregulation of *Foxp3* emerges as a potential mechanism for the regulation of IL-10 secretion by Treg lymphocytes. These findings are supported by the results of Moreno-Vilet et al. (17), who observed an upregulation of *Foxp3* induced by agavins from *Agave salmiana* in an *in vitro* model of peripheral blood mononuclear cells from healthy subjects. Similarly, the enrichment in *M. intestinale* and *B. uniformis* found in AG-fed mice also supports this potential mechanism, since both bacteria have been previously associated with an anti-inflammatory activity by stimulating Il-10 secretion (11, 52). Indeed, the upregulation of *Foxp3* has been correlated with the degradation of fiber and long-chain oligosaccharides by the gut microbiota. This is consistent with the increase in glycan-degrader bacterial taxa from the Bacteroidota phylum and a butyrate proportion observed in agavin-treated obese mice. In particular, butyrate has been shown to be involved in the promotion of colonic Treg generation and function by increasing the expression of the *Foxp3* transcription factor (63).

Dietary xenobiotics can also induce an anti-inflammatory response and the restoration of the intestinal epithelium during damage or disruption (64). For instance, diet rich in fruits and vegetables as well as polyphenols and their metabolites are recognized as natural ligands for the aryl hydrocarbon receptor (*Ahr*), a transcription factor involved in the maintenance and function of mucosal immune cells and regulation of gut inflammation. Even if CP alone did not upregulate *Ahr* in the present study, the combination of CP with AG, administered to obese mice (HFHS+CP+AG), resulted in a significant *Ahr* overexpression. Interestingly, Singh et al. (65) observed that mice fed the ellagitannin microbial metabolite urolithin A (Uro-A) induced the expression of *Ahr*, in conjunction with an upregulation of the epithelial tight-junction proteins through the activation of nuclear factor erythroid 2-related factor 2 (*AhR-Nrf2*). This was corroborated in the present study, as we observed that the *Ahr* upregulation in CP+AG-fed mice occurred along with the lowest levels of plasma LBP and significantly lower levels of *MyD88*. Whereas LBP and *MyD88* are implicated in TLR4-mediated signaling pathways involved in the activation of pro-inflammatory transcription factors, the low LPB levels and the downregulation of *Myd88* suggest a reinforcement of the intestinal epithelial barrier and a control of metabolic endotoxemia and inflammation (66). Moreover, modulation of gut microbiota by CP+AG could in turn improve the gut microbial dysbiosis and restore its symbiotic relationship with the host. We still do not know how agavins can synergize the effect of polyphenols on *Ahr* immune priming. We speculate that a greater amount of polyphenols reaching the colon as a result of a potential synergy with agavins would increase both the availability of polyphenols and the load of *Ahr*-agonists (35, 37, 42), signaling in turn this transcription factor.

While the present study provides insight on the mechanisms of polyphenols and oligosaccharides on the gut microbiota and the interaction with the host immune system, it has some limitations that will need to be addressed in future experiments. First, the study on the immunomodulating role of polyphenols and agavins was confined to the gene expression of innate and adaptive markers in colon tissues. The lack of more robust techniques, such as flow cytometry and metatranscriptomic analyses of intestinal and immune cells, limits our interpretation of the functional regulatory impact of prebiotics in mucosal and T-cell subsets in the colon and other intestinal sections. Furthermore, metagenomic data of microbial dysbiosis are warranted to obtain a complete overview of the functional profile of the gut microbiota induced by polyphenols and agavins, even though the inferred functions from Tax4Fun provided some indications that the supplements also modulate the gut microbiota metabolism. Finally, the absence of treatment effects on important cardiometabolic parameters might be explained by the relatively short duration of the experiment. Future work should consider extending the duration of the dietary intervention, increasing the doses, and assessing different proportions of agavins and polyphenols to enhance our understanding of the effect between diet, gut microbiome, and the host immune system.

Our data show that consumption of both CP and AG protects the gut barrier function and improves immunomodulation. Although the influence of AG appears to be restricted to mucosal adaptive immunity and related butyrate production, both prebiotic ingredients exert modulatory effects on the gut microbial composition to improve obesity-associated metabolic and inflammatory disturbances. We therefore propose that the combination of CP and AG prebiotic ingredients appears to be a good strategy to elicit a broader spectrum of beneficial effects to modulate gut microbiota and local inflammation and thus to counteract metabolic endotoxemia.

## Data availability statement

The datasets presented in this study can be found in online repositories. The names of the repository/repositories and accession number(s) can be found below: NCBI with BioProject ID PRJNA811638 (https://www.ncbi.nlm.nih.gov/bioproject/811638).

## Ethics statement

The animal study was reviewed and approved by Animal Care Committee of the Sainte-Justine hospital.

## Author contributions

YD, DR, GP, EL, and AM conceived the study. M-CR-D and MR carried out the animal experiments. M-CR-D performed the histomorphological analysis of colon tissues, the 16S rRNA sequence processing, analysis of the gut microbiota composition, and functional profiling. M-CR-D and A-SM-L performed the microarray analyses, M-CR-D analyzed the microarray data, and performed the statistical analysis of gut microbiota and mouse phenotypes. A-SM-L was involved in the SCFA analysis, PCR assay, and gene expression analysis. A-SM-L and M-CR-D analyzed and interpreted the data. A-SM-L, M-CR-D, YD, and HJ discussed and wrote this manuscript. M-CR-D and A-SM-L are equivalent main authors. All authors contributed to reviewing and editing the manuscript.

## Funding

This work was funded by a grant from the Ministère de l’Économie, de l’Innovation et des Exportations (MEIE, PSR-SIIRI-948) and the Consortium de Recherche et Innovations en Bioprocédés Industriels du Québec (CRIBIQ).

## Acknowledgments

We acknowledge the contribution of COLCIENCIAS for granting a PhD scholarship to M-CR-D and the contribution of CONACYT for granting a PhD scholarship to A-SM-L.

## Conflict of interest

The authors declare that the research was conducted in the absence of any commercial or financial relationships that could be construed as a potential conflict of interest.

## Publisher’s note

All claims expressed in this article are solely those of the authors and do not necessarily represent those of their affiliated organizations, or those of the publisher, the editors and the reviewers. Any product that may be evaluated in this article, or claim that may be made by its manufacturer, is not guaranteed or endorsed by the publisher.
