# Supplementary material for: Cranberry polyphenols and agave agavins impact gut immune response and microbiota composition while improving gut barrier function, inflammation, and glucose metabolism in mice fed an obesogenic diet
Source: Front Immunol. 2022 Aug 16;13:871080. doi: 10.3389/fimmu.2022.871080 (PMC9424773; doi:10.3389/fimmu.2022.871080)
Supplement: Supplementary file 1 [file DataSheet_1.pdf]

Supplementary Material

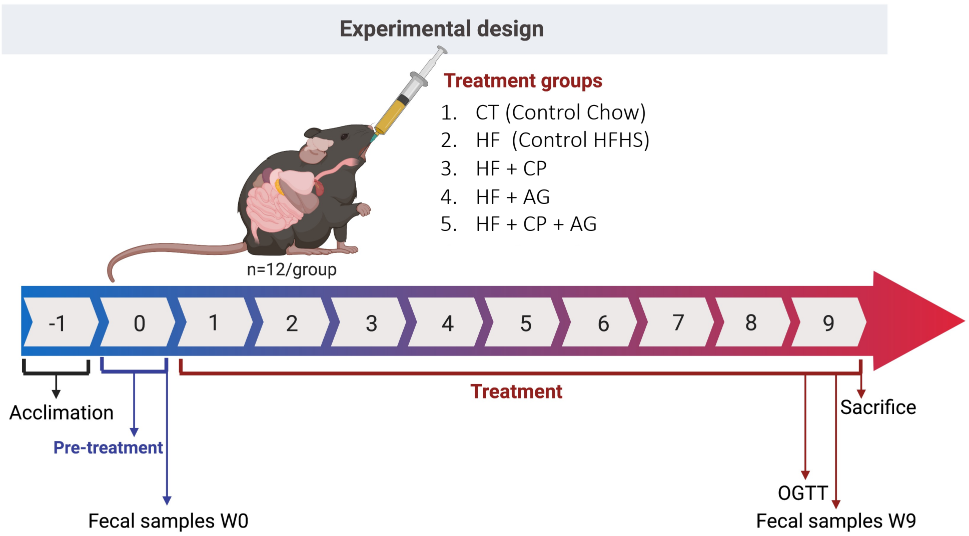

Figure S1. Experimental protocol. Sixty 6-weeks old C57BL/6J male mice, were randomly divided into five intervention groups (12 mice per group). Week -1 corresponded to acclimatation week, during this period all mice were fed a standard chow diet. Then, during week 0, mice were still fed a chow diet and pre-treated with the vehicle (water) (groups 1 and 2), or with their corresponding supplements, either with cranberry polyphenols alone (CP) (group 3), with agavins alone (AG) (group 4); or with both, cranberry polyphenols and agavins (CP+AG) (group 5). At the end of week 0, mice were assigned to their definitive diet as follows: group 1 continued chow diet, while groups 2, 3, 4 and 5 were fed a HFHS diet. Finally, for the intervention period, from week 1 to 9, mice received their corresponding diets and treatments for 9 weeks.

Table S1. Characterization of cranberry-polyphenols extract

**Phenolic content (mg/100 g dry weight)**	
**Polyphenol type**	**Whole Cranberry extract (CE)**
**Total phenolic content (%)**	**31.33% ± 1.01**
Anthocyanins	1628.2 ± 5.8
Cyanidin 3-galactoside	35.5 ± 1.8
Cyanidin 3-glucoside	602.8 ± 1.8
Cyanidin 3-arabinoside	403.6 ± 1.0
Peonidin 3-galactoside	108.3 ± 1.8
Peonidin 3-glucoside	460.5 ± 1.9
Peonidin 3-arabinoside	17.6 ± 2.2
**Proanthocyanins**	**10738.9 ± 100.8**
Monomers	998.5 ± 5.6
Dimers	2528.8 ± 28.9
Trimers	1567.5 ± 17.1
Tetramers	958.0 ± 2.3
Pentamers	433.4 ± 1.8
Hexamers	295.4 ± 7.0
Heptamers	89.7 ± 2.0
Octamers	68.7 ± 2.4
Nonamers	64.5 ± 1.0
Decamers	33.7 ± 3.1
Polymers >10	3700.8 ± 29.6
**Flavonols, flavan-3-nols and phenolic acids**	**4964.5**
Catechin	63.5 ± 5.3
Epicatechin	429.5 ± 12.8
Gallic acid	1.0 ± 0.3
Protocatechuic acid	275.3 ± 7.5
P-coumaric acid	1189.8 ± 49.4
Caffeic acid	139.4 ± 4.1
Ferulic acid	23.9 ± 4.9
3-caffeoylquinic acid	0.2 ± 0.1
4-caffeoylquinic acid	21.9 ± 1.8
5-caffeoylquinic acid	524.2 ± 6.8
Quercetine	831.3 ± 31.9
Quercetine-glucoside	629.7 ± 18.1
Quercetine-galactoside	ND
Quercetine-rhamnoside	376.4 ± 5.1
Quercetine-xyloside	378.8 ± 13.0
Quercetine-arabinoside	79.6 ± 7.8
Rutin	ND

Results are expressed as mean of triplicate ± SD. ND, not detected.

Table S2. Primer sequences used for RT-qPCR amplification

Gene	Sequences	Reference
Tnfα Forward	TAC TGA ACT TCG GGG TGA TTG GTC C	Lin et al, 2019 (1)
Tnfα Reverse	CAG CCT TGT CCC TTG AAG AGA ACC	
Il-1β Forward	TCG GAC CCA TAT GAG CTG A	Wang and Hatabu, 2019 (2)
Il-1β Reverse	CCA CAG GTA TTT TGT CGT TGC	
Nlrp6 Forward	TGA CCA GAG CTT CCA GGA GT	Wang and Hatabu, 2019 (2)
Nlrp6 Reverse	TTT AGC AGG CCA AAG AGG AA	
Ahr Forward	GGC TTT CAG CAG TCT GAT GTC	Lin et al, 2019 (1)
Ahr Reverse	CAT GAA AGA AGC GTT CTC TGG	
Tlr2 Forward	GCT GGA GGA CTC CTA GGC T	Wang et al., 2010 (3)
Tlr2 Reverse	GTC AGA AGG AAA CAG TCC GC	
Claudin-1 Forward	CTG GGT TTC ATC CTG GCT TC	Lin et al, 2019 (1)
Claudin-1 Reverse	TTG ATG GGG GTC AAG GGG T	
Muc2 Forward	GCC CGT GGA GTC GTA CGT GC	Lin et al, 2019 (1)
Muc2 Reverse	TTG GGG CAG AGT GAG GCG GT	
Myd88 Forward	TGG CCT TGT TAG ACC GTG A	Friedrich et al., 2017 (4)
Myd88 Reverse	AAG TAT TTC TGG CAG TCC TCC TC	
Actb Forward	CCA CCA TGT ACC CAG GCA TT	The present study
Actb Reverse	ACT CCT GCT TGC TGA TCC AC	
Ppib Forward	GGC ATG GAT GTG GTA CGG AA	The present study
Ppib Reverse	CCC CAG GCT CTC TAC TCC TT	
Gapdh Forward	CCA CAG TCC ATG CCA TCA CT	The present study
Gapdh Reverse	TAG GAA CAC GGA AGG CCA TG	
Hprt Forward	AGT CCC AGC GTC GTG ATT AG	The present study
Hprt Reverse	CCC CTT GAG CAC ACA GAG G	
Polr2b Forward	TCT CAC GTA TTC TGC TCC GC	The present study
Polr2b Reverse	TTC AGC TCA CAC AGG TCA CG	

1. Lin YH, Luck H, Khan S, Schneeberger PHH, Tsai S, Clemente-Casares X, Lei H, Leu YL, Chan YT, Chen HY, et al. Aryl hydrocarbon receptor agonist indigo protects against obesity-related insulin resistance through modulation of intestinal and metabolic tissue immunity. *Int J Obes* (2019) **43**:2407–2421. doi:10.1038/s41366-019-0340-1
2. Wang Y, Hatabu T. Mulberry juice freeze-dried powder attenuates the disease severity by the maintaining of colon mucosa in mice with DSS-induced acute colitis. *Biosci Biotechnol Biochem* (2019) **83**:914–922. doi:10.1080/09168451.2019.1580135
3. Wang Y, Devkota S, Musch MW, Jabri B, Nagler C, Antonopoulos DA, Chervonsky A, Chang EB. Regional Mucosa-Associated Microbiota Determine Physiological Expression of TLR2 and TLR4 in Murine Colon. *PLoS One* (2010) **5**:e13607. doi:10.1371/journal.pone.0013607
4. Friedrich C, Mamareli P, Thiemann S, Kruse F, Wang Z, Holzmann B, Strowig T, Sparwasser T, Lochner M. MyD88 signaling in dendritic cells and the intestinal epithelium controls immunity against intestinal infection with C. rodentium. *PLoS Pathog* (2017) **13**:e1006357. doi:10.1371/journal.ppat.1006357

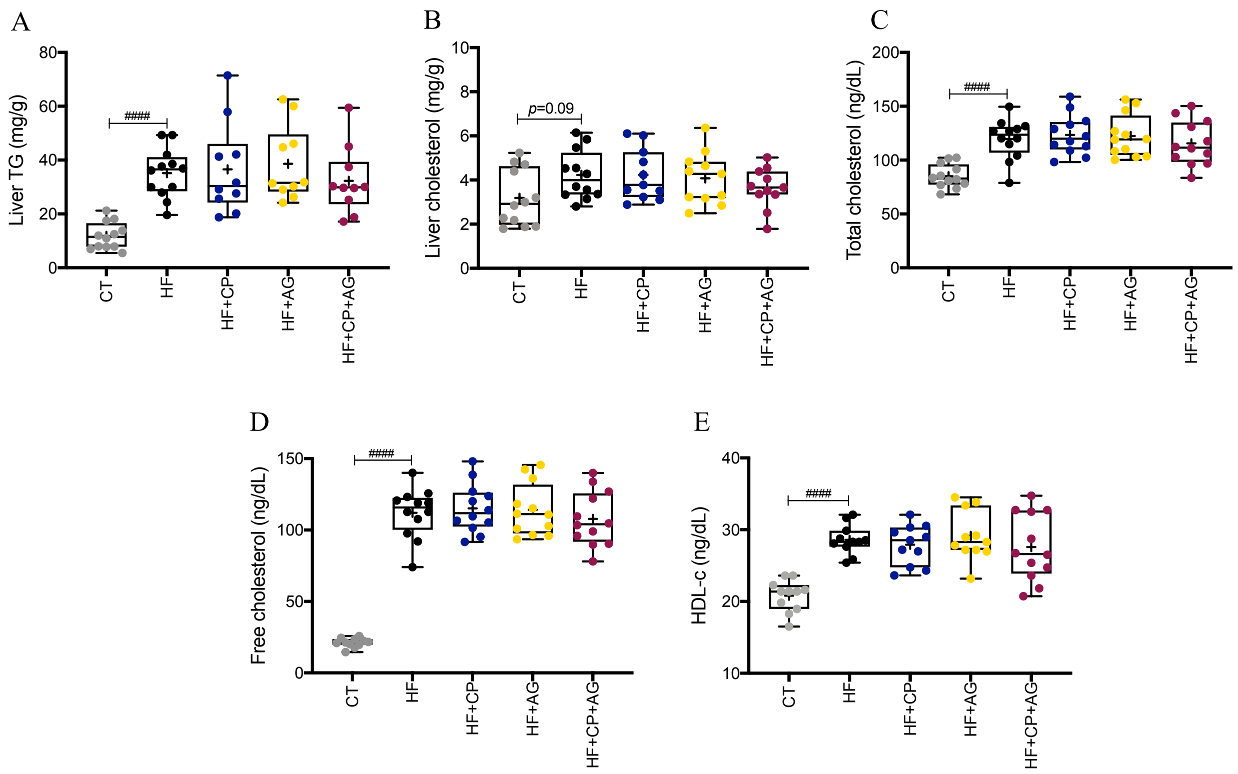

Figure S2. Effects of cranberry-polyphenols (CP) and agavins (AG) in lipid metabolism in HFHS-diet induced obese mice. Mice were fed either a Chow diet (CT) or High-fat high-sugar diet (HF); mice on a HF-diet were supplemented with CP (HF+CP), AG (HF+AG) or the combination of both CP+AG (HF+CP+AG) for 9 weeks. Blood samples were collected, and different parameters were measured **A)** Liver triglycerides level (mg/g); **B)** Liver cholesterol level (mg/g); **C)** Total cholesterol level (ng/dL); **D)** Free cholesterol level (ng/dL); **E)** High-density lipoprotein cholesterol levels (ng/dL). One-way ANOVA with a Dunnett's multiple comparison test (post hoc test) was employed to calculate the significance of the differences between groups. Values are expressed as the mean ± SEM. Boxplots represent the distribution of data with the mean represented by the mark "+" within the boxes, the median represented by the dark horizontal line and interquartile range by the box. #### p<0.0001 Chow-control group versus HFHS-control group.

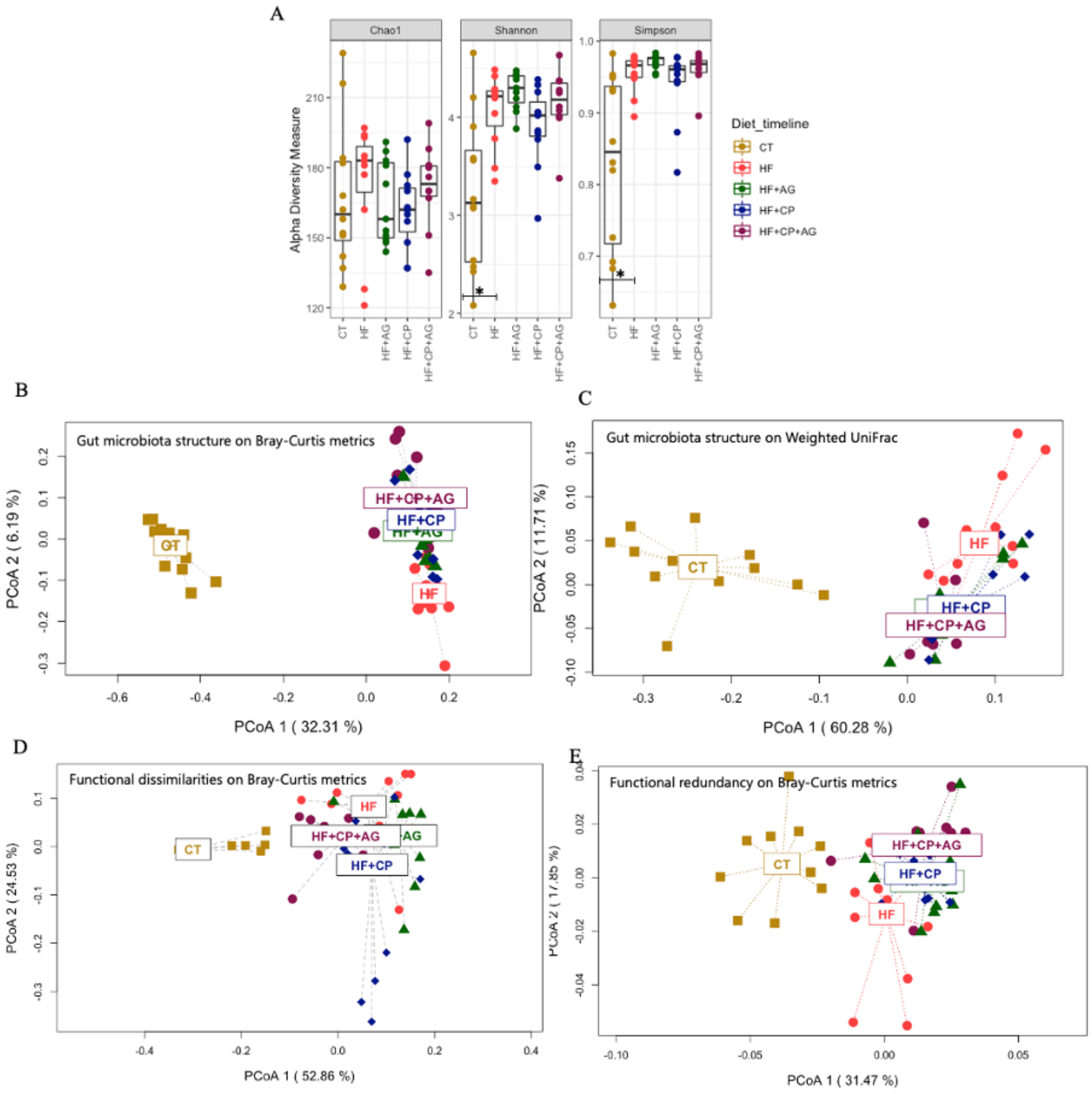

Figure S3. Effects of cranberry-polyphenols (CP) and agavins (AG) on the gut microbiota structure and predicted taxonomic functions in HFHS-diet induced obese mice. Mice were fed either a Chow diet (CT) or High-fat high-sugar diet (HF); mice on a HF-diet were supplemented with CP (HF+CP), AG (HF+AG) or the combination of both CP+AG (HF+CP+AG) for 9 weeks. Samples were collected for metagenomic analysis at the end of the intervention (week 9). **A)** α-diversity indices, determined by Chao1, Shannon-diversity and Simpson, was plotted for each dietary group at 9-weeks. Line inside the box represents the median, while whiskers represent the lowest and highest values within 1.5 interquartile range (IQR); **B)** Principal Coordinates Analysis (PCoA) plot-based Bray–Curtis metrics on taxonomic structure of samples at week 9 (PERMANOVA R^2 0.54452, p < 0.001); **C)** PCoA of taxon phylogenetic tree-based Weighted UniFrac of samples at week 9 (PERMANOVA R^2 0.60617 , p<0.001); **D)** PCoA plot of functional Bray–Curtis dissimilarities between samples at week 9 (PERMANOVA R^2 0.54452, p<0.001);

E)Functional redundancy-based PCoA plot (PERMANOVA R^2 0.35397 , $p < 0.001$). Corrected *p*-values having a tendency are indicated as "*q*". Each sample point (n = 12 per group) is color-coded based on the administered diet for 9-weeks, as shown in each figure legend.

A

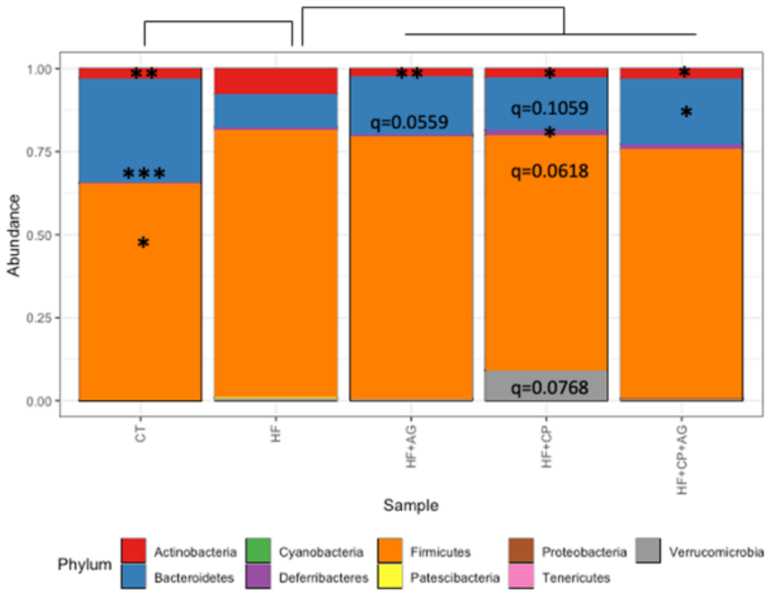

B

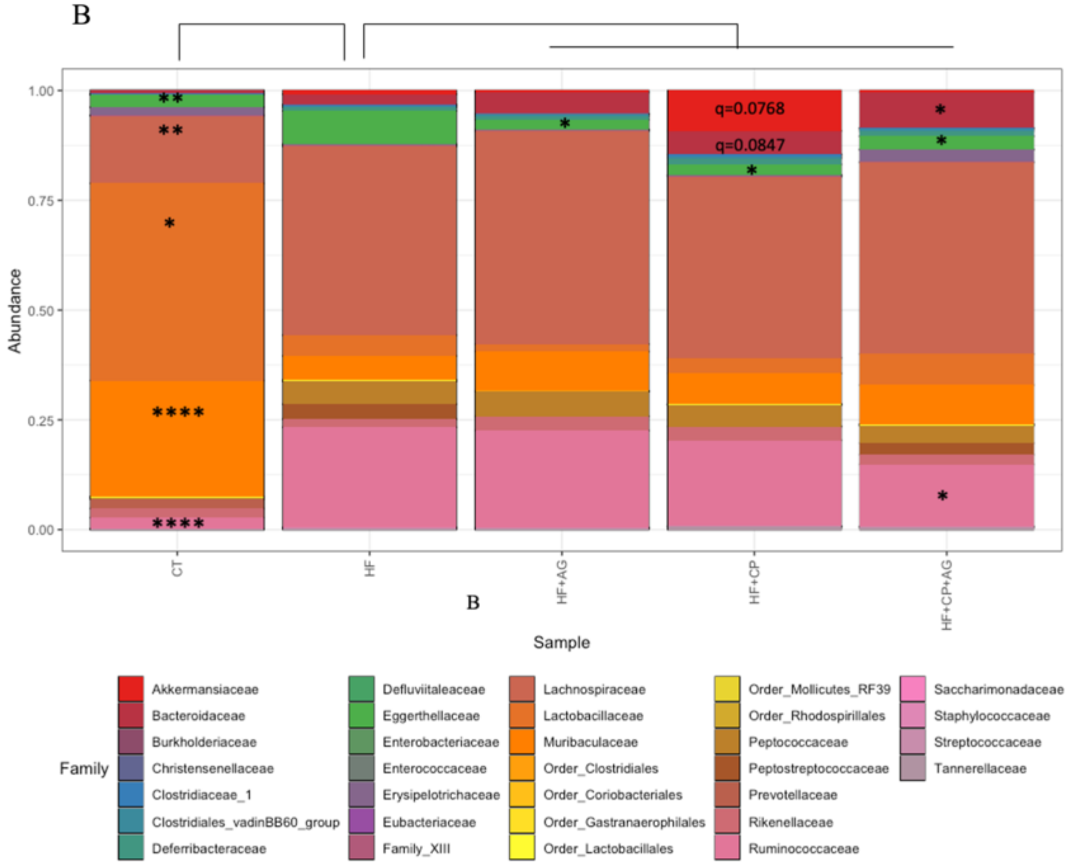

Figure S4 . Changes induced by cranberry-polyphenols and agavins on the relative proportions of bacterial taxa at the phylum and family level in the gut microbiota of HFHS-fed mice. CP selectively increased the relative abundance of Verrrucomicrobia phyla, CP+AG increased the

relative abundance of phylum Bacteroidetes (recently taxonomically named as Bacteroidota), and the relative abundance of Actinobacteria phyla was increased in all treated mice, as compared to HFHS-fed mice. **A)** Bar graph shows changes in the relative abundance of bacteria phyla at 9-weeks in mice fed HFHS (HF), Chow (CT), HFHS-diet supplemented with either cranberry-polyphenols (CP), agavins (AG), and cranberry-polyphenols with agavins (CP+AG); **B)** Bar graph of the relative abundances of bacterial families across the gut microbiota composition of each group. n=12 per group. Kruskal-Wallis test with FDR Benjamini and Hochberg post-hoc multiple comparison correction was performed to compare taxonomic abundance among groups. *$p<0.05$, **$p<0.01$, ***$p<0.001$, ****$p<0.0001$ as compared to HFHS-control group.

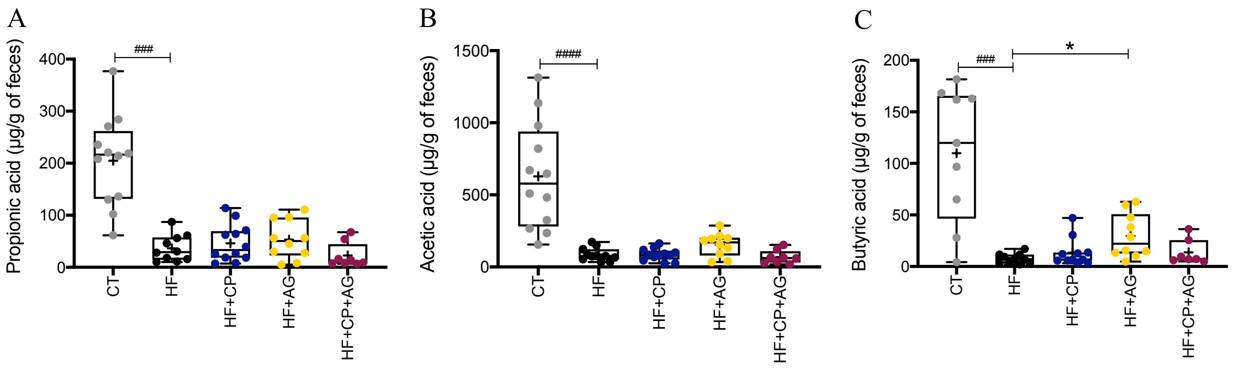

Figure S5. Cranberry-polyphenols and agavins distinctly modified the levels of short-chain fatty acids in HFHS-diet induced obese mice. Mice were fed either a Chow diet (CT) or High-fat high-sugar diet (HF); mice on a HF-diet were supplemented with CP (HF+CP), AG (HF+AG) or the combination of both CP+AG (HF+CP+AG) for 9 weeks. Fecal level of **A)** Propionic acid (µg/g of feces). **B)** Acetic acid (µg/g of feces). **C)** Butyric acid (µg/g of feces). One-way ANOVA with a Dunnett's multiple comparison test (post hoc test) was employed to calculate the significance of the differences between groups. Values are expressed as the mean ± SEM. Boxplots represent the distribution of data with the mean represented by the mark "+" within the boxes, the median represented by the dark horizontal line and interquartile range by the box. *$p < 0.05$ as compared to HFHS-control group. ###p<0.001 and ####p<0.0001 Chow-control group versus HFHS-control group.
